# Pre-Equilibrium Reaction Mechanism as a Strategy to Enhance Rate and Lower Overpotential in Electrocatalysis

**DOI:** 10.1021/jacs.2c10942

**Published:** 2023-02-03

**Authors:** Santanu Pattanayak, Louise A. Berben

**Affiliations:** Department of Chemistry, University of California, Davis, California, Davis, 95616, United States

## Abstract

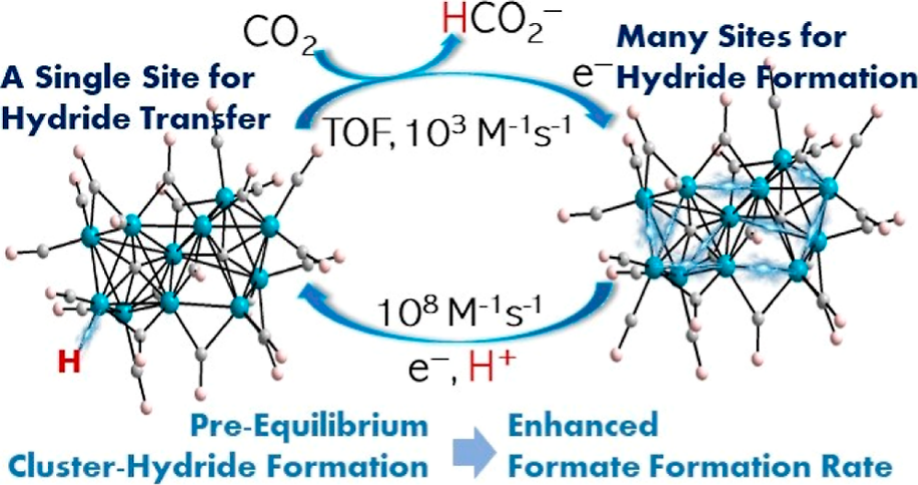

Pre-equilibrium reaction kinetics enable the overall rate of a catalytic reaction to be orders of magnitude faster than the rate-determining step. Herein, we demonstrate how pre-equilibrium kinetics can be applied to breaking the linear free-energy relationship (LFER) for electrocatalysis, leading to rate enhancement 5 orders of magnitude and lowering of overpotential to approximately thermoneutral. This approach is applied to pre-equilibrium formation of a metal-hydride intermediate to achieve fast formate formation rates from CO_2_ reduction without loss of selectivity (i.e., H_2_ evolution). Fast pre-equilibrium metal-hydride formation, at 10^8^ M^–1^ s^–1^, boosts the CO_2_ electroreduction to formate rate up to 296 s^–1^. Compared with molecular catalysts that have similar overpotential, this rate is enhanced by 5 orders of magnitude. As an alternative comparison, overpotential is lowered by ∼50 mV compared to catalysts with a similar rate. The principles elucidated here to obtain pre-equilibrium reaction kinetics via catalyst design are general. Design and development that builds on these principles should be possible in both molecular homogeneous and heterogeneous electrocatalysis.

## Introduction

**Scheme 1:**
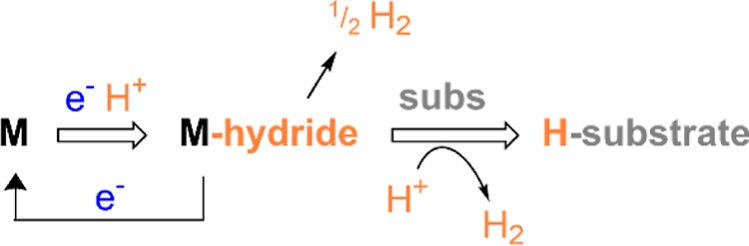
Three Common Pathways for the Metal–Hydride Reaction

Metal-hydrides are key intermediates in a broad scope of chemistries including solar fuels and organic transformations for commodity and fine chemical synthesis. However, electrocatalytic approaches involving metal-hydride intermediates are universally plagued by a competition between desired product formation (hydride transfer to substrate), and competitive reaction of the hydride intermediate with the protons that are needed in solution to generate the hydride intermediate [via electron-transfer (ET) and proton-transfer (PT) steps, Scheme 1].

An example situation where these competing reactions have been studied is in C–H bond formation with CO_2_ and the interest in that chemistry derives from its potential applications in solar fuel chemistry.^1−8^ Desired products include formate, methanol, ethanol, or ethylene which all contain C–H bonds; but H_2_ formation is an ongoing challenge. Contributions from a number of research groups have demonstrated that we can use catalyst design for *thermochemical* control of reaction chemistry to achieve selectivity for C–H bond formation over H_2_ evolution.^9−16^ However, that approach does not necessarily produce fast rates for C–H bond formation with CO_2_.^17^ Approaches to enhancing the reaction rate for C–H bond formation with CO_2_ are needed, and have primarily used reaction conditions, rather than catalyst design to achieve improvements: successful examples of this approach include stabilization of transition states for hydride transfer to CO_2_ by choice of the solvent,^18−25^ use of hydride-transfer mediators,^26^ or additions of base or alcohol.^27,28^

We have previously demonstrated that the large metal carbonyl clusters promote hydride formation with rate 10^8^ M^–1^ s^–1^.^29−31^ We believe that this fast rate has two possible origins. The multiple metal–metal bonds in the clusters serve as multiple sites for protonation, and this should provide a kinetic boost to the rate of cluster-hydride formation.^30,31^ In addition, the high anionic charges, 3– or 4–, on the clusters promote PT: while the delocalized structures of the clusters enable access to modest reduction potentials relative to single-metal site electrocatalysts, at a given formal oxidation state or overall charge.^32^

A remaining challenge is to design a complete catalytic cycle competent for solar fuel chemistry or chemical synthesis which has fast hydride formation without H_2_ production. Pre-equilibrium kinetic schemes have been reported as a tool for optimizing rates in O_2_ reduction,^33^ in hydride formation chemistries,^34^ and in CO_2_ reduction to CO.^35^ Pre-equilibrium dynamics of intermediate formation in a catalytic cycle often impact a subsequent rate. As an example, PT transfer rates to two-electron reduced [CoCp(dxpe)(NCCH_3_)]^2+^ complexes [Cp = cyclopentadienyl, dxpe = 1,2-bis(di(aryl/alkyl)phosphino)ethane] can be controlled by the equilibrium constant for dissociation of MeCN from Co prior to PT,^34^ and pre-equilibrium kinetics of CO_2_ binding to iron(0) porphyrin enhance the observed apparent rate constant for CO formation, under reaction conditions where the thermodynamics for C–O bond-breaking are favorable.^35^ Fast H_2_ evolution by iron porphyrin also proceeds with a pre-equilibrium kinetic scheme involving fast Fe-hydride formation.^36,37^

**Scheme 2:**
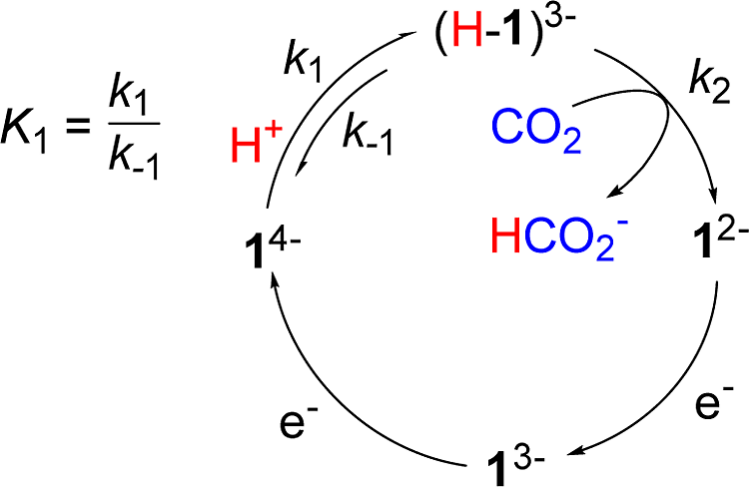
Proposed CO_2_ Reduction Mechanism by 1^3–^

When conceiving of the work reported herein, we reasoned that fast formation of (H-**1**)^3–^ at significant concentrations will boost the formate formation rate by orders of magnitude if (H-**1**)^3–^ is formed with very fast rate to enable a pre-equilibrium reaction mechanism. According to the pre-equilibrium approximation, which can be used in the case of a fast initial chemical step in a catalytic cycle, the observed rate of a possible formate formation reaction should scale with the equilibrium constant (*K*_1_) for pre-equilibrium hydride formation (Scheme 2), according to eq 1
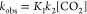
1where *k*_obs_ (s^–1^) is the observed rate of reaction, *K*_1_ is the equilibrium constant for formation of (H-**1**)^3–^, *k*_2_ (M^–1^ s^–1^) is the rate for hydride transfer to CO_2_, and [CO_2_] (M) is the concentration of CO_2_. We further noted during this experimental design that the fast pre-equilibrium chemical step should offer a kinetically derived lowering of the overpotential for the reaction because a fast chemical step following ET results in the anodic shift of the reduction peak potential of any electrocatalyst.

Herein, we demonstrate that formate is generated at an overpotential of 10 mV and with the rate of 1.2 × 10^3^ M^–1^ s^–1^ using **1**^3–^ in 0.1 M Bu_4_NBF_4_ MeCN/H_2_O (95:5) (Calculation S1). We can also use stronger acids than water to enhance *K*_1_, and with anisidinium tetrafluoro borate, it is generated at an overpotential of 64 mV with the rate of 5.07 × 10^2^ M^–1^ s^–1^. The well-understood mechanistic origin of this result from the pre-equilibrium mechanism provides a roadmap for future catalyst design. In principle, any electrocatalytic reaction can be designed with a view to achieving pre-equilibrium intermediate formation to overcome slow rates that might be associated with thermochemically controlled rates for selectivity in subsequent chemical steps using heterogeneous or homogeneous electrocatalysis.

## Results and Discussion

**Figure 1:**
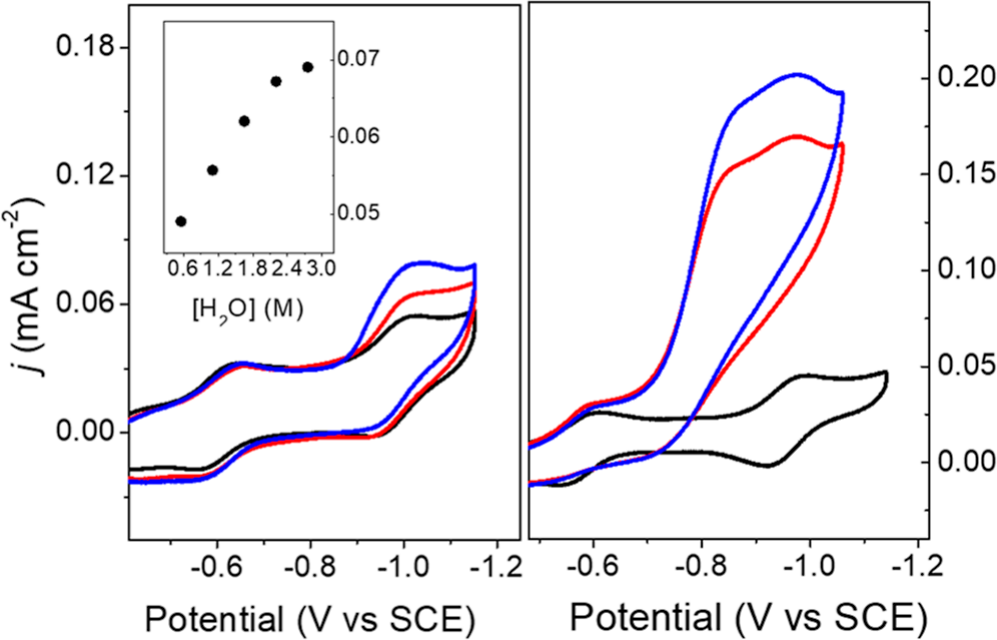
CV of 0.05 mM **1**^2–^ in 0.1 M Bu_4_NBF_4_ MeCN solution (black): (left) in 0.1 M Bu_4_NBF_4_ MeCN/H_2_O (95:5) under 1 atm N_2_ (red); in 0.1 M Bu_4_NBF_4_ MeCN/H_2_O (95:5) under 1 atm CO_2_ (blue). (right) with 5.1 mM AnsdH^+^ under 1 atm N_2_ (red) and with 0.25 mM AnsdH^+^ under 1 atm CO_2_ (blue). Inset: Plot of *j* versus [H_2_O] under 1 atm CO_2_.

To study catalysis by **1**^3–^, we prepared samples of (PhCH_2_NMe_3_)_2_[Co_11_C_2_(CO)_23_] (**1**^2–^) following a previously published method (PhCH_2_NMe_3_^+^ = benzyl ammonium cation).^38^ The CV of 0.05 mM **1**^2–^ recorded in 0.1 M Bu_4_NBF_4_ MeCN solution under 1 atm N_2_ shows three reversible redox couples with *E*_1/2_ = −0.2, −0.57, and −0.95 V versus SCE which were assigned as the **1**^1–/2–^, **1**^2–/3–^, and **1**^3–/4–^ couples, respectively (Figure S1).^31,38^ For experiments probing the catalytic activity of **1**^3–^, this species is generated in the CV measurement in all discussions below starting from **1**^2–^. When solutions of 0.05 mM **1**^2–^ in 0.1 M Bu_4_NBF_4_ MeCN were titrated with water, the current density, *j*_p_, at −1.054 V increased linearly up to 4% (2.2 M) H_2_O after which the changes in *j*_p_ were very small (Figure 1 left). This increase in *j*_p_ suggests that a catalytic reaction is occurring. CV of 0.05 mM **1**^2–^ collected under 1 atm CO_2_ in 0.1 M Bu_4_NBF_4_ MeCN/H_2_O (95:5) showed a further increase in *j*_p_ at −1.054 V, relative to the CV collected under 1 atm N_2_, and this suggests that a catalytic reaction has occurred where hydride is transferred to CO_2_ to afford formate (Figure 1 left). Current enhancements consistent with catalytic formate formation were also observed using anisidinium tetrafluoroborate (AnsdH^+^) as the source of H^+^ (Figure 1 right).

The most acidic proton source in solutions of CO_2_-saturated 0.1 M Bu_4_NBF_4_ MeCN/H_2_O (95:5) is carbonic acid produced from 0.24 M CO_2_ in MeCN/H_2_O and that has p*K*_a_ = 17.03.^39,40^ The p*K*_a_ of AnsdH^+^ in MeCN is 11.86.^41^ The waveforms obtained with MeCN/H_2_O (95:5) or with AnsdH^+^ are different. Catalysis with water is observed at similar potential as the reduction potential for **1**^3–/4–^, whereas catalysis using AnsdH^+^ as the proton source is observed at a potential more anodic than the reduction of **1**^3–^. A possible mechanistic origin of these waveforms is discussed later, along with the determination of *k*_obs_.

### Characterization of Formate

Controlled potential electrolysis (CPE) experiments carried out under both 1 atm N_2_ and 1 atm CO_2_ were performed to identify the product in the CV experiments. CPE experiments −1.13 V over 40 min were followed by analysis of the head space using gas chromatography with thermal conductivity detector (GC-TCD) and analysis of the solution using proton NMR spectroscopy. Using 5% H_2_O as the proton source in MeCN solutions, we determined that the Faradaic efficiency (FE) for formate and H_2_ production are 75(5) and 15(2) %, respectively (Table S1, Figures S2–S4, see the Supporting Information for experimental details). CPE experiments performed using AnsdH^+^ as the source of protons under 1 atm CO_2_ were run at −0.9 V, and those yielded formate and H_2_ with FE of 70(8) and 25(3)%, respectively (Table S1, Figures S2 and S3). No CO_2_-reduced products were detected by proton NMR when the CPE experiments were carried out under 1 atm N_2_ or in the absence of **1**^2–^ under 1 atm CO_2_. To confirm the carbon source, isotopically labelled ^13^CO_2_ was used for CPE experiments, and the ^13^C{^1^H} NMR spectrum collected of the CPE solution showed a peak at 172.9 ppm which conclusively indicates that formate was produced from CO_2_ during electrocatalysis (Figure S3D). CPE experiments run with the used electrodes from CPE experiments containing **1**^2–^, and those also produced no carbon-containing products. SEM–EDX measurements performed on used electrodes revealed no deposited Co on the glassy carbon (Figure S5).

### Mechanistic Studies of Hydride Formation

**Figure 2:**
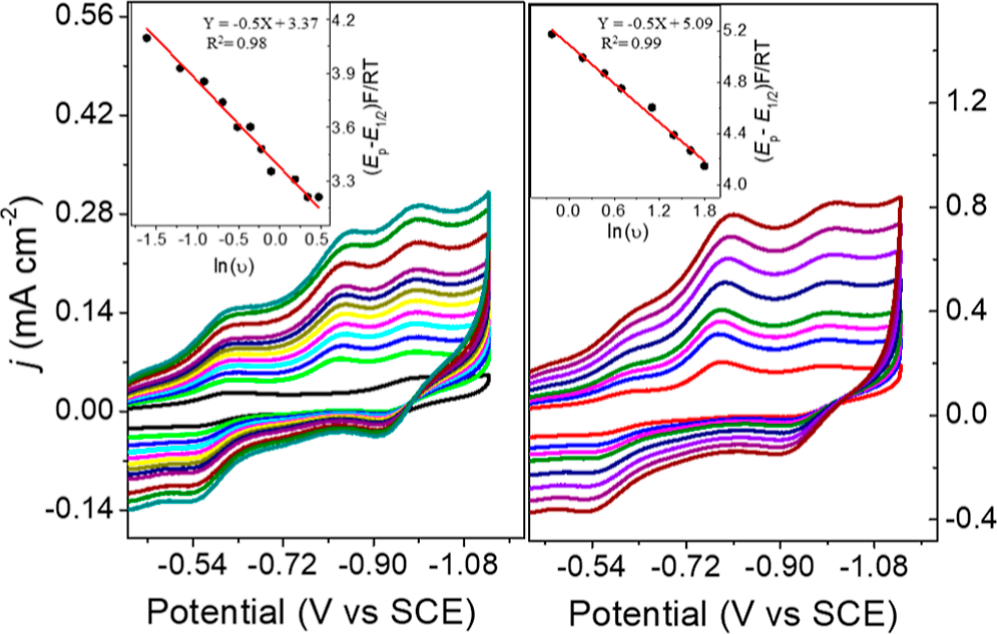
CVs of 0.1 mM **1**^2–^ in 0.1 M Bu_4_NBF_4_ MeCN/H_2_O (99.3:0.7). (left) Under 1 atm N_2_ at variable scan rates (starting from bright green 0.2, 0.3, 0.4, 0.5, 0.6, 0.7, 0.8, 0.9, 1.2, 1.4, and 1.6 V s^–1^); CV in black is for **1**^2–^ without added H_2_O. (right) Under 1 atm CO_2_ at variable scan rates (starting from red trace 0.8, 1.2, 1.6, 2, 3, 4, 5, and 6 V s^–1^). Insets: plots of (*E*_p_ – *E*_1/2_) (F/RT) vs ln(*υ*). The red line is a linear fit with slope fixed at −0.5.

Our first step toward understanding the mechanism for formate formation by **1**^3–^ was to measure the rate for catalyst-hydride, (H-**1**)^3–^, formation (*k*_1_, Scheme 2) in MeCN/H_2_O (95:5) under both N_2_ and CO_2_ atmospheres, where clusters of H_2_O–MeCN (under N_2_ and CO_2_) and/or carbonic acid (under CO_2_), respectively, are the proton sources for (H-**1**)^3–^ formation. Use of low [H^+^] in CV experiments accesses a kinetic region where the (H-**1**)^3–^ formation rate is measured based on the shift in peak potential: with low [H^+^], the follow-up chemical steps in the catalytic cycle are suppressed, and a small return wave is observed in the CV at ∼−0.72 V (Figure 2).

Accordingly, we collected CVs of 0.1 mM **1**^2–^ with 0.7% (0.38 mM) H_2_O in 0.1 M Bu_4_NBF_4_ MeCN under N_2_ and under CO_2_ at 100 mV/s (Figure 2). Under N_2_, the reductive peak potential *E*_p,c_ shifted from *E*_1/2_ anodically by 100 mV relative to CVs lacking H_2_O, which suggests a fast rate for PT following the ET. The **1**^3–/4–^ redox couple is also observed at a consistent potential in these CVs, which suggests that some of the **1**^2–^ is regenerated from (H-**1**)^3–^ in a reaction with protons during the CV experiment, and some of the (H-**1**)^3–^ remains and is oxidized at −0.72 V on the return scan.

For a chemical reaction that proceeds ET, the peak potentials (*E*_p_) shift anodically relative to the formal potential of **1**^3–/4–^ (*E*_1/2_). The kinetic information is contained in the “peak shift”, Δ*E*_p_ (where Δ*E*_p_ = *E*_p_ – *E*_1/2_) according to 2(42,43)
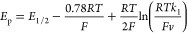
2where *R* is ideal gas constant (8.314 J mol^–1^*K*^–1^), *T* is temperature (*K*), *F* is Faraday’s constant (C mol^–1^), *k*_1_ is the second-order rate constant of the PT reaction (s^–1^), and other symbols were defined earlier. Due to the fast catalysis following the formation of (H-**1**)^3–^ (vide infra), eq 2 is potentially a little inaccurate in this situation, and therefore, we also determined a value for *k*_1_ using a foot-of-the wave analysis (FOWA),^44,45^ so that the two measurements can be compared.

Experimentally, *k*_1_ can be obtained by recording *E*_p_ – *E*_1/2_ as a function of *υ*, and we performed this experiment with a ratio of [H^+^]/[**1**^2–^] = 3800, where *E*_p_ – *E*_1/2_ is already ∼150 mV due to the fast PT step. A plot of (*E*_p_ – *E*_1/2_)F/RT vs ln(*υ*) according to eq 2 gave *k*_1_ = 3.9 × 10^5^ M^–1^ s^–1^ under 1 atm N_2_ (Table 1, Figure 2 left, Calculation S2). The same experiment was repeated under 1 atm CO_2_, and a higher rate of 1.2 × 10^7^ M^–1^ s^–1^ was observed for *k*_1_ (Table 1, Figure 2 right, Calculation S2). This higher rate for *k*_1_ is consistent with carbonic acid as the proton source, which has lower p*K*_a_ than H_2_O as the proton source in MeCN. The value of *k*_1_ measured using AnsdH^+^ as the proton source was the same (within error) under 1 atm of N_2_ or CO_2_ and is 3 × 10^8^ M^–1^ s^–1^ (Table 1, Figures 2, S6, Calculation S2). FOWA of CV traces collected under catalytic conditions yielded values for *k*_1_ that are in good agreement with the peak-shift analysis (Calculation S2, Table 1, Figure S7).

**Table 1:** Thermodynamic and Kinetic Data for (H-1)^3–^ Formation by 1^3–^a

[H^+^]/mM	p*K*_a_	*E*_pc_/V	*E*_pc/2_/V	*k*_1_^b^/M^–^^1^ s^–^^1^
H_2_O, N_2_ (380)		–0.85	0.80	3.9 × 10^5^(1.9 × 10^5^)^c^
H_2_O, CO_2_ (380)	17.03	–0.83	–0.78	1.2 × 10^7^(1 × 10^7^)^c^
AnsdH^+^, N_2_ (0.15)	11.8	–0.81	–0.76	3 × 10^8^^d^^,^^e^
AnsdH^+^, CO_2_ (0.25)	11.8	–0.81	–0.76	4.6 × 10^8^^e^

a Measured in 0.1 mM Bu_4_NBF_4_ MeCN with H_2_O or AnsdH^+^ under 1 atm CO_2_ or N_2_.

b *k*_1_ calculated using peak shift analysis (Calculation S2).

c Calculated using FOWA analysis (Calculation S2).

d Value from ref (31).

e Overlap of peak with **1**^2–/3–^ couple precluded FOWA.

### Catalytic Formate Formation Rate and Mechanism

**Figure 3:**
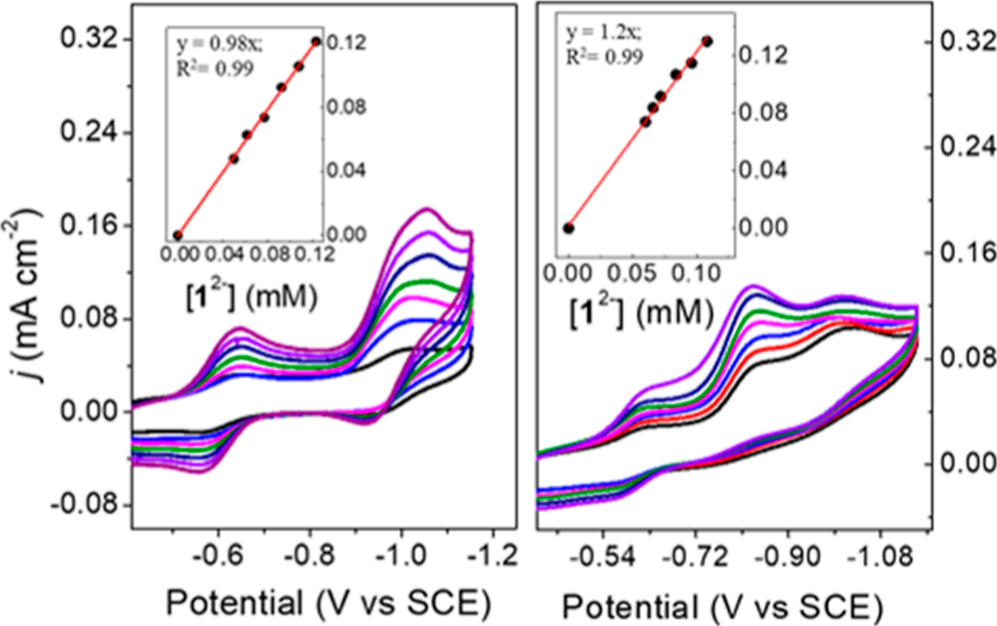
CVs of **1**^2–^ with (0.05, 0.06, 0.77, 0.09, 0.11-, and 0.12 mM H^+^, colors) in 0.1 M Bu_4_NBF_4_ MeCN under CO_2_, at 100 mV/s and using the GC electrode. (left) with 5% H_2_O as the source of H^+^. Inset: plot of *j*_c_ versus [**1**^2–^], at −1.054 V. (right) with 2 mM AnsdH^+^ as the source of H^+^. Inset: plot of *j*_c_ vs [**1**^2–^], at −0.82 V. Red lines are linear fit to the data, and black CV trace has no added H^+^.

Our next effort toward understanding the effects of pre-equilibrium kinetics on formate production by **1**^2–^ was to characterize the chemistry under reaction conditions that promote turnover in the catalytic cycle. CV experiments were performed with varied [**1**^2–^] in 0.1 M Bu_4_NBF_4_ MeCN under CO_2_, and the acid source was either 5% (2.77 M) H_2_O or 2 mM AnsdH^+^ (Figure 3). In each case, a linear correlation between *j*_c_ versus [**1**^2–^] was observed, and this indicates that formate production is of first order in [**1**^2–^]. Similarly, the reaction is of first order with respect to [H^+^] under 1 atm CO_2_ when either H_2_O or AnsdH^+^ is the source of H^+^ (Figure S8).

**Figure 4:**
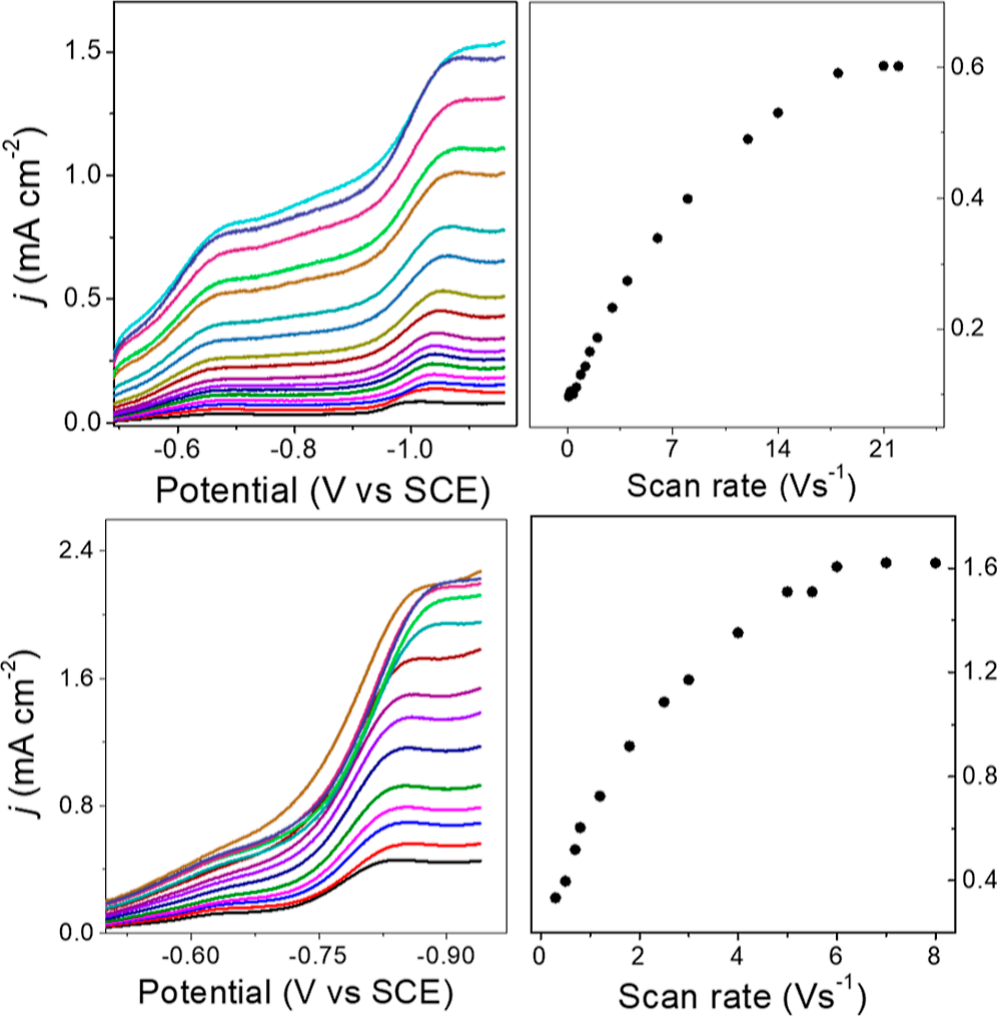
Forward CV traces of (top left) 0.06 mM **1**^2–^ in 0.1 M Bu_4_NBF_4_ MeCN/H_2_O (95:5) under 1 atm CO_2_ at the scan rate = 0.1, 0.2, 0.4, 0.6, 0.9, 1.2, 1.5, 2, 3, 4, 6, 8, 12, 14, 18, 21, and 22 V/s; and (bottom left) 3.5 mM AnsdH^+^ and 0.2 mM **1**^2–^ in 0.1 M Bu_4_NBF_4_ MeCN under CO_2_ at various scan rates: 0.3, 0.5. 0.7, 0.8, 1.2, 1.8, 2.5, 3, 4, 5, 5.5, 6, 7, and 8 V/s. (Right) Plots of *j*_max_ vs υ at potentials negative to −1.08 V (top) and −0.82 (bottom), after subtraction of the background current value.

The rate of formate formation by **1**^2–^ (*k*_obs_, s^–1^) can be obtained using a fast scan CV measurement of the limiting current (*i*_lim_).^46,47^ The experiment is performed with excess substrate relative to the catalyst to achieve *i*_lim_, which is independent of the scan rate due to mutual compensation of substrate depletion during catalysis and diffusion. These reaction conditions also lead to the form of eq 1, where neither [**1**^3–^] or [H^+^] influences *k*_obs_. Analysis of *i*_lim_ to measure the rate of formate formation under 1 atm CO_2_ was performed using the same two sources of protons as mentioned above: CO_2_-saturated H_2_O and AnsdH^+^, in 0.1 M Bu_4_NBF_4_ MeCN solution (Calculation S4, Figure 4). In addition, the *k*_obs_ values obtained were corrected for the measured FE which are 75 and 70% for formate under 1 atm CO_2_, in MeCN/H_2_O (95:5) and MeCN with added AnsdH^+^, respectively. Therefore, the values for *k*_obs_ are 296 and 142 s^–1^, respectively, in MeCN/H_2_O (95:5) and MeCN with added AnsdH^+^, under 1 atm CO_2_ (Table 1).

A comparison of the data collected with low [H^+^] (Figure 2) and with high [H^+^] for catalytic conditions (Figure 4) provides some information about the mechanism for formate formation with either H_2_O or AnsdH^+^ and how those mechanisms may differ slightly as a function of the proton source. As described earlier, under reaction conditions with low [H^+^], there is an anodic shift in *E*_p,c_(**1**^3–/4–^) due to the folfast PT reaction which affords (H-**1**)^3–^ from **1**^4–^. The anodic shift in *E*_p,c_(**1**^3–/4–^) from *E*_1/2_ is 140 and 100 mV with H_2_O or AnsdH^+^, respectively, under 1 atm CO_2_ (Table 1). As the [H^+^] increases, the catalytic current response with H_2_O gradually shifts cathodically, whereas the response under AnsdH^+^ remains at *E*_cat/2_ = −0.78 V (Table 2). Both of these behaviors are common in molecular electrocatalysis and indicate nuances in the reaction mechanism. With AnsdH^+^, the constant value of *E*_cat/2_ over a wide range for [H^+^] simply suggests that the mechanism for formate formation (or background H_2_ evolution) is unchanged even as the concentration of (H-**1**)^3–^ available in solution increases with increased [H^+^] (Scheme 2). When H_2_O/carbonic acid is the proton source, then at higher [H^+^], *E*_cat/2_ for **1**^2–^ shifts cathodically so that *E*_cat/2_ ∼ *E*_1/2_ at [H^+^]_max_. Water (or carbonic acid) is a weaker acid than AnsdH^+^ by 5 p*K*_a_ units. The cathodic shift in *E*_cat/2_ with increased [H^+^] may therefore arise from a competing bimolecular evolution of H_2_ as has been described in prior reports by us^31^ and by others (Scheme 1).^48^ It is also possible that more negative potentials are needed to drive hydride transfer from (H-**1**)^3–^ to CO_2_ under the solution conditions containing 5% H_2_O; perhaps due to lower solubility of CO_2_, CO_2_ equilibria with H_2_CO_3_ and HCO_3_^–^, or relative solvation of the substrate in solution.

**Table 2:** Thermodynamic and Kinetic Data for Formate Formation by 1^3–^a

[H^+^]/mM	*E*_cat/2_/V	*K*_1_^b^	*k*_obs_^c^/s^–1^	*k*_cat_^c^/M^–1^ s^–1^
H_2_O, CO_2_ (2800)	–1.01	15.8	296	1.2 × 10^3^
AnsdH^+^, CO_2_ (3.8)	–0.78	5.5 × 10^12^	142	5.1 × 10^2^

a Measured in 0.1 mM Bu_4_NBF_4_ MeCN with excess H_2_O (carbonic acid) or AnsdH^+^, under 1 atm CO_2_. *E*_1/2_ (**1**^3–/4–^) = −0.95 V versus SCE.

b See Calculation S3 for *K*_1_.

c *k*_obs_ obtained from the fast scan method (Calculation S4); values are corrected for the FE of formate (Table S1).

### Pre-equilibrium Effects on Catalysis

The formate formation rate catalyzed by **1**^2–^ (*k*_obs_) is orders of magnitude slower than the rate for formation of the intermediate (H-**1**)^3–^ (*k*_1_) (vide supra). Therefore, the formation of (H-**1**)^3–^ can be considered as a pre-equilibrium step with both a forward and reverse rate constant, *k*_1_ and *k*_–1_, and an equilibrium constant, *K*_1_ = *k*_1_/*k*_–1_ (Scheme 2). A key feature of the pre-equilibrium mechanism is that *k*_obs_ is enhanced linearly according to the magnitude of *K*_1_: that is a “normal” mechanism would have *k*_obs_ = *k*_2_[CO_2_], but the pre-equilibrium mechanism has *k*_obs_ = *K*_1_*k*_2_[CO_2_] (eq 1, and the terms and units were defined earlier).

Even at very low ratios of [H^+^]/[**1**^2–^] under 1 atm CO_2_, the **1**^3–/4–^ redox couple is irreversible, and therefore, we cannot determine *K*_1_ using an electrochemical measurement (Figure 3 right).^49^ However, we can estimate a value for *K*_1_ if we know the p*K*_a_ value for (H-**1**)^3–^ and the p*K*_a_ values for the proton sources used for catalysis, which are CO_2_ saturated H_2_O and AnsdH^+^. We determined the p*K*_a_ value for (H-**1**)^3–^ as 24.6 using infra-red spectroelectrochemical titrations of **1**^4–^ with acid sources (Calculation S5, Figure S9). We then used thermochemical cycles to determine the values for *K*_1_ from the p*K*_a_ values of (H-**1**)^3–^, CO_2_ saturated water, and AnsdH^+^ (Calculation S3). The values of *K*_1_ were determined as 15.8 and 5.5 × 10^12^, when CO_2_ saturated water or AnsdH^+^ are used as the proton source, respectively.^50−52^ Using the experimentally determined value for *k*_1_, this calculation further provides an estimate of *k*_–1_ as 1.2 × 10^4^ and 5.4 × 10^–5^ M^–1^ s^–1^, when CO_2_ saturated water or AnsdH^+^ are used as the proton source, respectively, since *K*_1_ = *k*_1_/*k*_–1_. According to these estimates, *K*_1_ > 1, and we should expect that the pre-equilibrium formation of (H-**1**)^3–^ enhances *k*_obs_ relative to *k*_2_ (eq 1, Scheme 2).

### Catalyst Benchmarking

**Figure 5:**
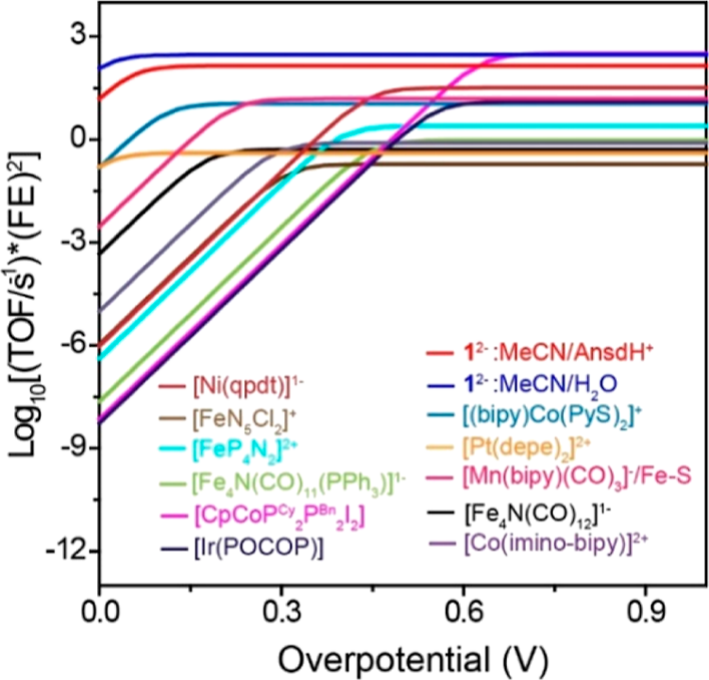
Tafel-style plot: Log_10_[(TOF/s^–1^) (EF)^2^] vs overpotential (*η*) at *E*_cat/2_, for selected molecular CO_2_ to HCO_2_^–^ reduction catalysts. Details of calculations and parameters used to construct the plot are shown in Table S2 and references therein. *η* = *E*_CO2/HCOO^–^_ – *E*_cat/2_.

As mentioned in the introduction, we predicted that two features of the catalyst performance (rate and overpotential) will be enhanced by the pre-equilibrium reaction kinetics, relative to reports of formate formation by other molecular catalysts. These two effects are nicely illustrated using a Tafel style plot where the TOF (which is equivalent to *k*_obs_ above) is plotted as log_10_(TOF/s^–1^) versus overpotential (V). On this plot, formate formation by **1**^3–^ is illustrated using both AnsdH^+^ and H_2_O as the source of protons (Figure 5). Overpotential is defined as
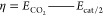
3where η is overpotential (mV), *E*_CO_2__ is the thermodynamic potential for reduction of CO_2_ into formate under standard conditions (mV), and *E*_cat/2_ is the potential at which the catalytic current density reaches half of its maximum current (*i*_cat/2_ and see Calculation S1).^53^

Kinetic enhancements to *k*_1_ result in a low overpotential for the catalytic reaction at 64 mV using **1**^3–^ in MeCN/AnsdH^+^. Specifically, *E*_1/2_ for **1**^3–^ is −0.95 V, but *E*_cat/2_ is −0.78 V, and the 170 mV anodic shift in *E*_cat/2_ has a kinetic origin in the extremely fast PT rate and formation of (H-**1**)^3–^, at 3 × 10^8^ M^–1^ s^–1^. Another example of a very fast formate forming catalyst is [(bipy)Co(PyS)_2_]^+^ which has TOF similar to **1**^3–^ while the overpotential remains pinned to the value of *E*_1/2_ so that the overpotential for formate formation is 110 mV.^54^

**Figure 6:**
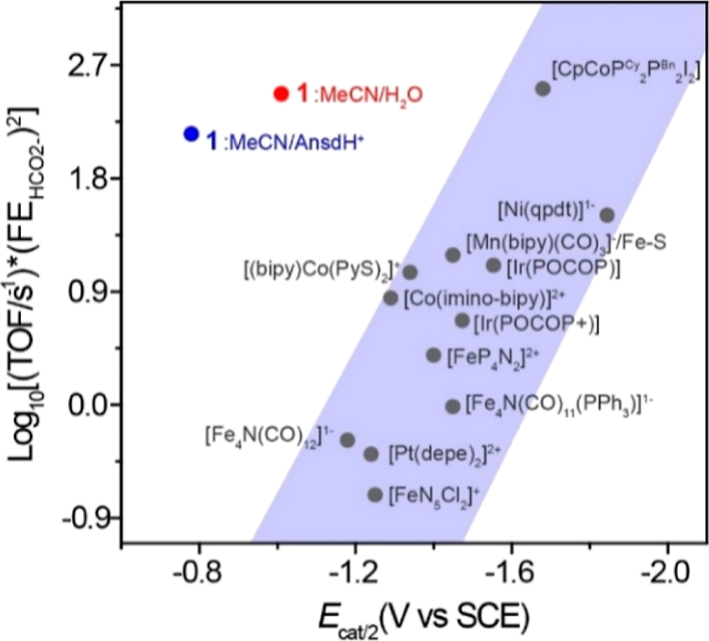
LFER between Log(TOF/s^–1^) and *E*_cat/2_ for selected molecular electrocatalysts {**1**^2–^, [Fe_4_N(CO)_12_]^1–^,^20,25^ [Fe_4_N(CO)_11_(PPh_3_)]^−^,^56^ [FeN_5_Cl_2_]^+^,^57^ [FeP_4_N_2_]^2+^,^58^ [Co(imino-bpy)]^2+^,^59^ [(bipy)Co(PyS)_2_]^+^,^54^ CpCoP^Cy^_2_N^Bn^_2_I_2_,^18^ [Ni(qpdt)_2_]^−^,^60^ [Pt(depe)_2_]^+^,^15,16^ Ir(POCOP),^22^ and [Mn(bipy)(CO)_3_]/Fe–S}.^26^ Data for this plot, see Table S2. The blue shadow highlights correlation of Log_10_[(TOF/s^–1^)(FE_HCOO^–^_)^2^] with *E*_cat/2_.

A plot of Log_10_(TOF/s^–1^) versus *E*_cat/2_ for molecular catalysis of CO_2_ to formate shows a linear correlation (Figure 6). Absent kinetic effects, a linear free-energy relationship (LFER) should exist between Log(TOF/s^–1^) and hydricity (free energy for loss of hydride, Δ*G*_H^–^_) over a series of catalysts.^55^ It is also known that Δ*G*_H^–^_ scales roughly with *E*_cat/2_ and p*K*_a_ of the catalytic hydride intermediate over a series of catalysts having similar mechanism, and this relationship underlies a rough correlation between Log_10_(TOF/s^–1^) and *E*_cat/2_. Given that the structure of **1**^3–^ is different than the structure of the single-site metal catalysts that comprise most of the catalysts in Figures 5 and 6, it is possible that **1**^3–^ does not fall on the Δ*G*_H^–^_ versus *E*_cat/2_ correlation line for those compounds. Therefore, we include a brief discussion using Δ*G*_H^–^_ as a benchmark against related catalysts to complement the observations in Figure 6.

To assess the driving force for hydride transfer from (H-**1**)^3–^ to CO_2_, we first determined Δ*G*_H^–^_ for (H-**1**)^3–^. The Δ*G*_H-_ was obtained from the p*K*_a_ for (H-**1**)^3–^ using a thermochemical cycle, and it is 41.1 ± 2.6 kcal mol^–1^ in MeCN when using an organic acid as the source of protons (Calculation S5, Figure S9).^61−64^ The Δ*G*_H^–^_ for formate in MeCN is 44 kcal mol^–1^,^11^ and that suggests that reaction of (H-**1**)^3–^ with CO_2_ to give **1**^2–^ and HCOO^–^ is favorable by 2.9 kcal mol^–1^ which is relatively close to thermoneutral. Other example catalysts which have hydricity close to thermoneutral for C–H bond formation with CO_2_ include [Fe_4_N(CO)_11_(PPh_3_)]^−^,^56^ Pt(dmpe)_2_,^14^ and [(bipy)Co(PyS)_2_]^+^,^52^ which have Δ*G*_H^–^_ estimated at 45, 41.4, and 38 kcal mol^–1^, respectively. We note that the hydricity for [H-Fe_4_N(CO)_11_(PPh_3_)]^−^ and [H–Pt(dmpe)_2_] was determined experimentally, and for [(bipy)Co(PyS)_2_]^+^, a theoretical estimate was made.^14,54,56^ The catalysts **1**^3–^ and [Pt(dmpe)_2_]^2+^ have near-identical hydricity in MeCN but **1**^3–^ produces formate with a rate that is 5 orders of magnitude faster as a result of the pre-equilibrium reaction mechanism. Another direct comparison involves [(bipy)Co(PyS)_2_]^+^, which has hydricity 3.1 kcal mol^–1^ stronger than **1**^3–^, and yet, the two catalyst exhibit inverted rates: 1.2 × 10^3^ M^–1^ s^–1^ for **1**^3–^(65,66) and 98.2 M^–1^ s^–1^ for [(bipy)Co(PyS)_2_]^+^ in MeCN, which we also attribute to the pre-equilibrium kinetics of (H-**1**)^3–^ formation.

### Catalyst Design for a Pre-Equilibrium Mechanism

These findings illustrate how clusters, or more generally nano-sized materials with delocalized electronic structure, can be employed to enhance the reaction rate of the first chemical step in the catalytic cycle to achieve the pre-equilibrium mechanism. The active catalyst, **1**^3–^, has 3- charge while retaining a very modest reduction potential of −0.95 V. The low potential, despite the high anionic molecular charge, likely arises from low reorganization energy associated with the delocalized bonding in the metal–metal bonded cluster; the fast PT to afford (H-**1**)^3–^ may be promoted by the high anionic charge on intermediate **1**^4–^, by the large array of almost-identical surface sites that are available to react with the proton, and by a low reorganization energy for the PT. In any electrochemically driven catalytic cycle, a fast chemical step following ET will lower the overpotential for catalysis, as defined in eq 3, since *E*_cat/2_ is kinetically shifted by the fast chemical step.

Using the cluster structure to achieve a pre-equilibrium mechanism has further advantages beyond the lowered overpotential described in the preceding paragraph. Following generation of the intermediate, in this case (H-**1**)^3–^, there is now just one reactive site on the catalytic intermediate, and thus, the selectivity of the second chemical step remains under thermochemical control with a rate that is enhanced by the pre-equilibrium value of *K*_1_ according to eq 1.

*Regarding future applications* of the pre-equilibrium mechanism to enhance the electrocatalyst performance, there are a few obvious scenarios that come to mind. The establishment of the pre-equilibrium depends on the reactivity of both the catalyst (more precisely, the catalyst following a redox event) and the substrate that is required for intermediate formation. These will be discussed separately. Regarding the catalyst: heterogeneous and nano-scale catalysts possess delocalized structures and multiple reactive surface sites similar to the clusters described herein and should be amenable to design of pre-equilibrium mechanisms for solar fuel chemistry. Molecular catalysts, likewise, are promising candidates, and proton relays are a structure type in this category that is well-known to be effective in fast and low overpotential H2 evolution from protons. Beyond, proton reduction, new strategies in molecular chemistry must provide multiple sites for specific substrate binding or site specificity, and possible ideas in this area include incorporation of H-bond accepting or -donating functional groups that are chosen with a p*K*_a_ value that is not suitable for proton relay behavior. A nucleophilic molecular catalyst is another obvious approach, but that is not a good one since it also results in high energy (very cathodic potentials in the case of reduction) for the catalytic turnover.

Regarding the substrate: in this work, the substrate needed for intermediate formation was a proton, and the proton activity is easily changed (and benchmarked according to the p*K*_a_ scale) to promote a large equilibrium constant (*K*_1_: see eq 1, Scheme 2). The tuning of substrates such as CO_2_, CO, or N_2_ may be a little more challenging, but strategies are known which can drive fast catalyst/substrate interactions that have high equilibrium constants. As examples, Lewis acid co-catalysts are known to polarize overall non-polar molecules including N_2_ and CO_2_, just like the anion associated with a proton changes its p*K*_a_ value and activity. Other mechanisms for tuning the reactivity of substrates include use of heterogeneous or homogeneous electrocatalysts with chemically inequivalent bind sites to polarize incoming substrates, to serve as ET sites, and to stabilize intermediates and enhance *K*_1_.

It is apparent from the foregoing discussion that many of the catalyst design strategies reported by researchers in the heterogeneous and homogeneous electrocatalysis and solar fuel communities may already be drawing on pre-equilibrium reaction mechanisms to achieve high performance through tuning of the catalyst, substrate, or both. Recognition of those pre-equilibrium mechanisms will result in better control and further enhanced performance because it can guide tuning of elementary steps in the catalytic cycles. Alternatively, minor adjustments to the substrate choice or catalyst design may induce pre-equilibrium mechanisms from existing catalytic cycles that involve successive ET and chemical reaction steps.

## Conclusions

In this report, we described a general strategy for use of the pre-equilibrium reaction mechanism to enhance the reaction rate. This was illustrated for formate formation from CO_2_ and catalyzed by [Co_11_C_2_(CO)_23_]^3–^ (**1**^3–^). Relative to the known LFER for Log_10_(TOF/s^–1^) versus *E*_cat/2_ for reported catalysts, the reaction rate to form formate was enhanced by 5 orders of magnitude, and the overpotential was lowered by 100 mV. Specific to the example demonstrated herein, pre-equilibrium metal-hydride formation led to the enhanced catalyst performance. Selectivity for formate formation (over H_2_ formation or other CO_2_ reduction products) arises from the thermoneutral hydride transfer elementary chemical step, whereas pre-equilibrium kinetic effects originate in the hydride formation at 3 × 10^8^ M^–1^ s^–1^. A rationale for the observed rates and selectivity were discussed in this report, in relation to the nano-scale structure of **1**^3–^ and the choice of the proton source which both promote the pre-equilibrium reaction mechanism. In addition, the generality and clearly understood origin of the effects presented herein can be applied broadly to the design of homogeneous and heterogeneous catalysts, and possible strategies to achieve this in future efforts were discussed.

## Supporting Information Available

Experimental methods, calculations, CPE results, CV measurements, and details of electrochemical analysis (PDF)

## References

[ref1] Nitopi S.; Bertheussen E.; Scott S. B.; Liu X.; Engstfeld A. K.; Horch S.; Seger B.; Stephens I. E. L.; Chan K.; Hahn C.; Nørskov J. K.; Jaramillo T. F.; Chorkendorff I. Progress and Perspectives of Electrochemical CO_2_ Reduction on Copper in Aqueous Electrolyte. Chem. Rev. 2019, 119, 7610–7672. 10.1021/acs.chemrev.8b00705. 31117420

[ref2] Olah G. A.; Goeppert A.; Prakash G. K. Chemical Recycling of Carbon Dioxide to Methanol and Dimethyl Ether: From Greenhouse Gas to Renewable, Environmentally Carbon Neutral Fuels and Synthetic Hydrocarbons. J. Org. Chem. 2009, 74, 487–498. 10.1021/jo801260f. 19063591

[ref3] Olah G. A.; Goeppert A.; Surya Prakash G. K. Beyond Oil and Gas: The Methanol Economy, 2nd ed.; Wiley-VCH: Weinheim, Germany, 2009; pp 233–288.

[ref4] Chen H.; Dong F.; Minteer S. D. The progress and outlook of bioelectrocatalysis for the production of chemicals, fuels and materials. Nat. Catal. 2020, 3, 225–244. 10.1038/s41929-019-0408-2.

[ref5] De Luna P.; Hahn C.; Higgins D.; Jaffer S. A.; Jaramillo T. F.; Sargent E. H. What would it take for renewably powered electrosynthesis to displace petrochemical processes?. Science 2019, 364, eaav3506 10.1126/science.aav3506. 31023896

[ref6] Appel A. M.; Bercaw J. E.; Bocarsly A. B.; Dobbek H.; DuBois D. L.; Dupuis M.; Ferry J. G.; Fujita E.; Hille R.; Kenis P. J.; Kerfeld C. A.; Morris R. H.; Peden C. H.; Portis A. R.; Ragsdale S. W.; Rauchfuss T. B.; Reek J. N.; Seefeldt L. C.; Thauer R. K.; Waldrop G. L. Frontiers, opportunities, and challenges in biochemical and chemical catalysis of CO_2_ fixation. Chem. Rev. 2013, 113, 6621–6658. 10.1021/cr300463y. 23767781 PMC3895110

[ref7] Inglis J. L.; MacLean B. J.; Pryce M. T.; Vos J. G. Electrocatalytic Pathways towards Sustainable Fuel Production from Water and CO_2_. Coord. Chem. Rev. 2012, 256, 2571–2600. 10.1016/j.ccr.2012.05.002.

[ref8] Ross M. B.; De Luna P.; Li Y.; Dinh C.-T.; Kim D.; Yang P.; Sargent E. H. Designing materials for electrochemical carbon dioxide recycling. Nat. Catal. 2019, 2, 648–658. 10.1038/s41929-019-0306-7.

[ref9] Taheri A.; Berben L. A. Tailoring Electrocatalysts for Selective CO_2_ or H^+^ Reduction: Iron Carbonyl Clusters as a Case Study. Inorg. Chem. 2016, 55, 378–385. 10.1021/acs.inorgchem.5b02293. 26689238

[ref10] Loewen N. D.; Neelakantan T. V.; Berben L. A. Renewable Formate from C-H Bond Formation with CO_2_: Using Iron Carbonyl Clusters as Electrocatalysts. Acc. Chem. Res. 2017, 50, 2362–2370. 10.1021/acs.accounts.7b00302. 28836757

[ref11] Waldie K. M.; Ostericher A. L.; Reineke M. H.; Sasayama A. F.; Kubiak C. P. Hydricity of Transition-Metal Hydrides: Thermodynamic Considerations for CO_2_ Reduction. ACS Catal. 2018, 8, 1313–1324. 10.1021/acscatal.7b03396.

[ref12] Ceballos B. M.; Yang J. Y. Directing the reactivity of metal hydrides for selective CO_2_ reduction. Proc. Natl. Acad. Sci. U.S.A. 2018, 115, 12686–12691. 10.1073/pnas.1811396115. 30463952 PMC6294940

[ref13] Yang J.; Kerr T.; Wang X.; Barlow J. Reducing CO_2_ to HCO_2_^–^ at Mild Potentials: Lessons from Formate Dehydrogenase. J. Am. Chem. Soc. 2020, 142, 19438–19445. 10.1021/jacs.0c07965. 33141560

[ref14] Barlow J. M.; Yang J. Y. Thermodynamic Considerations for Optimizing Selective CO_2_ Reduction by Molecular Catalysts. ACS Cent. Sci. 2019, 5, 580–588. 10.1021/acscentsci.9b00095. 31041377 PMC6487447

[ref15] Cunningham D. W.; Barlow J. M.; Velazquez R. S.; Yang J. Y. Reversible and Selective CO_2_ to HCO_2_^–^ Electrocatalysis near the Thermodynamic Potential. Angew. Chem., Int. Ed. 2020, 59, 4443–4447. 10.1002/anie.201913198. 31846551

[ref16] Cunningham D. W.; Yang J. Y. Kinetic and Mechanistic Analysis of a Synthetic Reversible CO_2_/HCO_2_^–^ Electrocatalyst. Chem. Commun. 2020, 56, 12965–12968. 10.1039/d0cc05556e. 32996485

[ref17] Fogeron T.; Todorova T. K.; Porcher J. P.; Gomez-Mingot M.; Chamoreau L. M.; Mellot-Draznieks C.; Li Y.; Fontecave M. A Bioinspired Nickel(Bis-Dithiolene) Complex as a Homogeneous Catalyst for Carbon Dioxide Electroreduction. ACS Catal. 2018, 8, 2030–2038. 10.1021/acscatal.7b03383.

[ref18] Roy S.; Sharma B.; Pécaut J.; Simon P.; Fontecave M.; Tran P. D.; Derat E.; Artero V. Molecular Cobalt Complexes with Pendant Amines for Selective Electrocatalytic Reduction of Carbon Dioxide to Formic Acid. J. Am. Chem. Soc. 2017, 139, 3685–3696. 10.1021/jacs.6b11474. 28206761

[ref19] Loewen N. D.; Berben L. A. Secondary Coordination Sphere Design to Modify Transport of Protons and CO_2_. Inorg. Chem. 2019, 58, 16849–16857. 10.1021/acs.inorgchem.9b03102. 31802660

[ref20] Taheri A.; Carr C. R.; Berben L. A. Electrochemical Methods for Assessing Kinetic Factors in the Reduction of CO_2_ to Formate: Implications for Improving Electrocatalyst Design. ACS Catal. 2018, 8, 5787–5793. 10.1021/acscatal.8b01799.

[ref21] Sullivan B. P.; Meyer T. J. Kinetics and Mechanism of Carbon Dioxide Insertion into a Metal-Hydride Bond. A Large Solvent Effect and an Inverse Kinetic Isotope Effect. Organometallics 1986, 5, 1500–1502. 10.1021/om00138a035.

[ref22] Kang P.; Cheng C.; Chen Z.; Schauer C. K.; Meyer T. J.; Brookhart M. Selective Electrocatalytic Reduction of CO_2_ to Formate by Water-Stable Iridium Dihydride Pincer Complexes. J. Am. Chem. Soc. 2012, 134, 5500–5503. 10.1021/ja300543s. 22390391

[ref23] Kang P.; Meyer T. J.; Brookhart M. Selective Electrocatalytic Reduction of Carbon Dioxide to Formate by a Water-Soluble Iridium Pincer Catalyst. Chem. Sci. 2013, 4, 3497–3502. 10.1039/c3sc51339d. 24899071

[ref24] Mayberry D. D.; Linehan J. C.; Appel A. M. Designing Catalytic Systems Using Binary Solvent Mixtures: Impact of Mole Fraction of Water on Hydride Transfer. Inorg. Chem. 2021, 60, 17132–17140. 10.1021/acs.inorgchem.1c02397. 34723498

[ref25] Taheri A.; Thompson E. J.; Fettinger J. C.; Berben L. A. An Iron Electrocatalyst for Selective Reduction of CO_2_ to Formate in Water: Including Thermochemical Insights. ACS Catal. 2015, 5, 7140–7151. 10.1021/acscatal.5b01708.

[ref26] Dey S.; Masero F.; Brack E.; Fontecave M.; Mougel V. Electrocatalytic Metal Hydride Generation Using Concerted Proton Electron Transfer Mediators. Nature 2022, 607, 499–506. 10.1038/s41586-022-04874-z. 35859199

[ref27] Madsen M. R.; Rønne M. H.; Heuschen M.; Golo D.; Ahlquist M. S. G.; Skrydstrup T.; Pedersen S. U.; Daasbjerg K. Promoting Selective Generation of Formic Acid from CO_2_ Using Mn(Bpy)(CO)_3_Br as Electrocatalyst and Triethylamine/Isopropanol as Additives. J. Am. Chem. Soc. 2021, 143, 20491–20500. 10.1021/jacs.1c10805. 34813304

[ref28] Bhattacharya M.; Sebghati S.; VanderLinden R. T.; Saouma C. T. Toward Combined Carbon Capture and Recycling: Addition of an Amine Alters Product Selectivity from CO to Formic Acid in Manganese Catalyzed Reduction of CO_2_. J. Am. Chem. Soc. 2020, 142, 17589–17597. 10.1021/jacs.0c07763. 32955864 PMC7584391

[ref29] Cesari C.; Shon J. H.; Zacchini S.; Berben L. A. Metal Carbonyl Clusters of Groups 8-10: Synthesis and Catalysis. Chem. Soc. Rev. 2021, 50, 9503–9539. 10.1039/d1cs00161b. 34259674

[ref30] Carr C. R.; Taheri A.; Berben L. A. Fast Proton Transfer and Hydrogen Evolution Reactivity Mediated by [Co_13_C_2_(CO)_24_]^4-^. J. Am. Chem. Soc. 2020, 142, 12299–12305. 10.1021/jacs.0c04034. 32571013

[ref31] Pattanayak S.; Berben L. A. Cobalt Carbonyl Clusters Enable Independent Control of Two Proton Transfer Rates in the Mechanism for Hydrogen Evolution. ChemElectroChem 2021, 8, 2488–2494. 10.1002/celc.202100402.

[ref32] Helm M. L.; Stewart M. P.; Bullock R. B.; DuBois M. R.; DuBois D. L. A Synthetic Nickel Electrocatalyst with a Turnover Frequency Above 100,000 s^–1^ for H_2_. Science 2011, 333, 863–866. 10.1126/science.1205864. 21836012

[ref33] Pegis M. L.; Martin D. J.; Wise C. F.; Brezny A. C.; Johnson S. I.; Johnson L. E.; Kumar N.; Raugei S.; Mayer J. M. Mechanism of Catalytic O2 Reduction by Iron Tetraphenylporphyrin. J. Am. Chem. Soc. 2020, 141, 8315–8326. 10.1021/jacs.9b02640. PMC6684231 31042028

[ref34] Kurtz D. A.; Dhar D.; Elgrishi B.; Kandemir S. F.; McWilliams W. C.; Howland C.; Chen J. L.; Dempsey J. L. Redox-Induced Structural Reorganization Dictates Kinetics of Cobalt(III) Hydride Formation via Proton-Coupled Electron Transfer. J. Am. Chem. Soc. 2021, 143, 3393–3406. 10.1021/jacs.0c11992. 33621088

[ref35] Costentin C.; Drouet S.; Passard G.; Robert M.; Savéant J. M. Proton-Coupled Electron Transfer Cleavage of Heavy-Atom Bonds in Electrocatalytic Processes. Cleavage of a C-O Bond in the Catalyzed Electrochemical Reduction of CO2. J. Am. Chem. Soc. 2013, 135, 9023–9031. 10.1021/ja4030148. 23692448

[ref36] Costentin C.; Savéant J. M. Homogeneous Catalysis of Electrochemical Reactions: The Steady-State and Nonsteady-State Statuses of Intermediates. ACS Catal. 2018, 8, 5286–5297. 10.1021/acscatal.8b01195.

[ref37] Costentin C.; Dridi H.; Savéant J. M. Molecular Catalysis of H2 Evolution: Diagnosing Heterolytic versus Homolytic Pathways. J. Am. Chem. Soc. 2014, 136, 13727–13734. 10.1021/ja505845t. 25190347

[ref38] Ciabatti I.; Femoni C.; Hayatifar M.; Iapalucci M. C.; Longoni G.; Pinzino C.; Solmi M. V.; Zacchini S. The Redox Chemistry of [Co_6_C(CO)_15_]^2-^: A Synthetic Route to New Co-Carbide Carbonyl Clusters. Inorg. Chem. 2014, 53, 3818–3831. 10.1021/ic500161e. 24654982

[ref39] Azcarate I.; Costentin C.; Robert M.; Savéant J. M. Dissection of Electronic Substituent Effects in Multielectron-Multistep Molecular Catalysis. Electrochemical CO_2_-to-CO Conversion Catalyzed by Iron Porphyrins. J. Phys. Chem. C 2016, 120, 28951–28960. 10.1021/acs.jpcc.6b09947.

[ref40] Costentin C.; Drouet S.; Robert M.; Savéant J.-M. A Local Proton Source Enhances CO2 Electroreduction to CO by a Molecular Fe Catalyst. Science 2012, 338, 90–94. 10.1126/science.1224581. 23042890

[ref41] Roberts J. A. S.; Bullock R. M. Direct Determination of Equilibrium Potentials for Hydrogen Oxidation/Production by Open Circuit Potential Measurements in Acetonitrile. Inorg. Chem. 2013, 52, 3823–3835. 10.1021/ic302461q. 23488870

[ref42] Savéant J.-M. Elements of Molecular and Biomolecular Electrochemistry; John Wiley & Sons: Hoboken, 2006; pp 78–93.

[ref43] Lee K. J.; Elgrishi N.; Kandemir B.; Dempsey J. L. Electrochemical and Spectroscopic Methods for Evaluating Molecular Electrocatalysts. Nat. Rev. Chem. 2017, 1, 0039 10.1038/s41570-017-0039.

[ref44] Wang V. C. C.; Johnson B. A. Interpreting the Electrocatalytic Voltammetry of Homogeneous Catalysts by the Foot of the Wave Analysis and Its Wider Implications. ACS Catal. 2019, 9, 7109–7123. 10.1021/acscatal.9b00850.

[ref45] Costentin C.; Savéant J.-M. Multielectron, Multistep Molecular Catalysis of Electrochemical Reactions: Benchmarking of Homogeneous Catalysts. ChemElectroChem 2014, 1, 1226–1236. 10.1002/celc.201300263.

[ref46] Azcarate I.; Costentin C.; Robert M.; Savéant J. M. Through-Space Charge Interaction Substituent Effects in Molecular Catalysis Leading to the Design of the Most Efficient Catalyst of CO_2_-to-CO Electrochemical Conversion. J. Am. Chem. Soc. 2016, 138, 16639–16644. 10.1021/jacs.6b07014. 27976580

[ref47] Cometto C.; Chen L.; Anxolabéhère-Mallart E.; Fave C.; Lau T.-C.; Robert M. Molecular Electrochemical Catalysis of the CO_2_-to-CO Conversion with a Co Complex: A Cyclic Voltammetry Mechanistic Investigation. Organometallics 2019, 38, 1280–1285. 10.1021/acs.organomet.8b00555.

[ref48] Costentin C.; Dridi H.; Savéant J. M. Molecular Catalysis of H_2_ Evolution: Diagnosing Heterolytic versus Homolytic Pathways. J. Am. Chem. Soc. 2014, 136, 13727–13734. 10.1021/ja505845t. 25190347

[ref49] Rountree E. S.; McCarthy B. D.; Dempsey J. L. Decoding Proton-Coupled Electron Transfer with Potential–pK_a_ Diagrams: Applications to Catalysis. Inorg. Chem. 2019, 58, 6647–6658. 10.1021/acs.inorgchem.8b03368. 31033279

[ref50] Rhile I. J.; Mayer J. M. One-Electron Oxidation of a Hydrogen-Bonded Phenol Occurs by Concerted Proton-Coupled Electron Transfer. J. Am. Chem. Soc. 2004, 126, 12718–12719. 10.1021/ja031583q. 15469234

[ref51] Schrauben J. N.; Cattaneo M.; Day T. C.; Tenderholt A. L.; Mayer J. M. Multiple-Site Concerted Proton-Electron Transfer Reactions of Hydrogen-Bonded Phenols Are Nonadiabatic and Well Described by Semiclassical Marcus Theory. J. Am. Chem. Soc. 2012, 134, 16635–16645. 10.1021/ja305668h. 22974135 PMC3476473

[ref52] Qiu G.; Knowles R. R. Rate-Driving Force Relationships in the Multisite Proton-Coupled Electron Transfer Activation of Ketones. J. Am. Chem. Soc. 2019, 141, 2721–2730. 10.1021/jacs.8b13451. 30665301 PMC6508549

[ref53] Appel A. M.; Helm M. L. Determining the Overpotential for a Molecular Electrocatalyst. ACS Catal. 2014, 4, 630–633. 10.1021/cs401013v.

[ref54] Dey S.; Todorova T. K.; Fontecave M.; Mougel V. Electroreduction of CO_2_ to Formate with Low Overpotential Using Cobalt Pyridine Thiolate Complexes. Angew. Chem. 2020, 132, 15856–15863. 10.1002/ange.202006269. 32673413

[ref55] Jeletic M. S.; Hulley E. B.; Helm M. L.; Mock M. T.; Appel A. M.; Wiedner E. S.; Linehan J. C. Understanding the Relationship between Kinetics and Thermodynamics in CO_2_ Hydrogenation Catalysis. ACS Catal. 2017, 7, 6008–6017. 10.1021/acscatal.7b01673.

[ref56] Loewen N. D.; Thompson E. J.; Kagan M.; Banales C. L.; Myers T. W.; Fettinger J. C.; Berben L. A. A Pendant Proton Shuttle on [Fe_4_N(CO)_12_]^-^ Alters Product Selectivity in Formate vs. H_2_ Production via the Hydride [H-Fe_4_N(CO)_12_]^-^. Chem. Sci. 2016, 7, 2728–2735. 10.1039/c5sc03169a. 28660048 PMC5477009

[ref57] Chen L.; Guo Z.; Wei X.; Gallenkamp C.; Bonin J.; Anxolabéhère-Mallart K.; Lau T.; Lau M.; Robert M. Molecular Catalysis of the Electrochemical and Photochemical Reduction of CO_2_ with Earth-Abundant Metal Complexes. Selective Production of CO vs HCOOH by Switching of the Metal Center. J. Am. Chem. Soc. 2015, 137, 10918–10921. 10.1021/jacs.5b06535. 26267016

[ref58] Bi J.; Hou P.; Liu F.; Kang P. Electrocatalytic Reduction of CO_2_ to Methanol by Iron Tetradentate Phosphine Complex Through Amidation Strategy. ChemSusChem 2019, 12, 2195–2201. 10.1002/cssc.201802929. 31050182

[ref59] Liu F.; Bi J.; Sun Y.; Luo S.; Kang P. Cobalt Complex with Redox-Active Imino Bipyridyl Ligand for Electrocatalytic Reduction of Carbon Dioxide to Formate. ChemSusChem 2018, 11, 1656–1663. 10.1002/cssc.201800136. 29577653

[ref60] Fogeron T.; Todorova T. K.; Porcher J.; Gomez-Mingot M.; Chamoreau L.; Mellot-Draznieks C.; Li Y.; Fontecave M. A Bioinspired Nickel(Bis-Dithiolene) Complex as a Homogeneous Catalyst for Carbon Dioxide Electroreduction. ACS Catal. 2018, 8, 2030–2038. 10.1021/acscatal.7b03383.

[ref61] Curtis C. J.; Miedaner A.; Ellis W. W.; DuBois D. L. Measurement of the Hydride Donor Abilities of [HM(Diphosphine)_2_]^+^ Complexes (M = Ni, Pt) by Heterolytic Activation of Hydrogen. J. Am. Chem. Soc. 2002, 124, 1918–1925. 10.1021/ja0116829. 11866604

[ref62] Matsubara Y.; Fujita E.; Doherty M. D.; Muckerman J. T.; Creutz C. Thermodynamic and Kinetic Hydricity of Ruthenium(II) Hydride Complexes. J. Am. Chem. Soc. 2012, 134, 15743–15757. 10.1021/ja302937q. 22966971

[ref63] Wiedner E. S.; Chambers M. B.; Pitman C. L.; Bullock R. M.; Miller A. J. M.; Appel A. M. Thermodynamic Hydricity of Transition Metal Hydrides. Chem. Rev. 2016, 116, 8655–8692. 10.1021/acs.chemrev.6b00168. 27483171

[ref64] The theoretical framework developed by Kubiak and coworkers,^11^ estimates hydricity for (H-**1**)^3–^ as 49.8 kcal mol^–1^ in MeCN solution (Calculation S6), but this assumes that bond dissociation free energy (BDFE) of metal hydride bonds varies only within ±10 kcal mol^–1^, and is likely not a good approximation for the delocalized and fluctional bonding in clusters.

[ref65] k_cat_ is calculated from the TOF and [CO2] = 0.24 M in MeCN/H2O or 0.28 M in MeCN,^66^ using k_obs_ = K_1_k_2_[CO2], where k_cat_ = K_1_k_2_.

[ref66] Tomita Y.; Teruya S.; Koga O.; Hori Y. Electrochemical Reduction of Carbon Dioxide at a Platinum Electrode in Acetonitrile-Water Mixtures. J. Electrochem. Soc. 2000, 147, 4164–4167. 10.1149/1.1394035.

