# Supplementary material for: Pre-Equilibrium Reaction Mechanism as a Strategy to Enhance Rate and Lower Overpotential in Electrocatalysis
Source: J Am Chem Soc. 2023 Feb 3;145(6):3419–26. doi: 10.1021/jacs.2c10942 (PMC9936576; doi:10.1021/jacs.2c10942)
Supplement: Supplementary file 1 — ja2c10942_si_001.pdf [file ja2c10942_si_001.pdf]

Supporting Information to accompany:

Pre-equilibrium Reaction Mechanism as a Strategy to Enhance Rate and Lower Overpotential in Electrocatalysis

Santanu Pattanayak and Louise A. Berben*

Department of Chemistry, University of California Davis, CA 95616, USA

Corresponding Author email: laberben@ucdavis.edu

Table of Contents.

1. Experimental Methods

2. Calculations

Calculation S1. Calculation of overpotential for CO_2 reduction to formate.

Calculation S2. Peak Shift and foot-of-the-wave analysis for hydride formation rate (k_1) calculation.

Calculation S3. Calculation of K_1 from pK_a values.

Calculation S4. Fast scan method for k_{obs} measurements

Calculation S5. Experimental determination of pK_a and hydricity for (H-**1**)$^{3-}$.

Calculation S6. Theoretical hydricity value estimation.

3. Tables

Table S1. Parameter to calculate Faradic Efficiency and Turnover number during CPE experiments.

Table S2. Kinetics and thermodynamic parameters used to construct Tafel plot for selected CO_2 reduction catalyst.

4. Figures

Figure S1. CV of 0.3 mM **1**$^{2-}$ recorded in 0.1 M Bu_4NBF_4 MeCN solution.

Figure S2. Charge vs. time plots from CPE performed in various conditions.

Figure S3. Calibration curve used to quantify H_2 based on the GC-TCD data.^{1}H NMR spectrum of CPE solution and calibration curve used to quantify formate. ^{13}C NMR spectrum of formate derived from $^{13}CO_2$.

Figure S4. IR spectra of **1**$^{2-}$ in 0.1 M Bu_4NBF_4 MeCN of a CPE solution before (black line) and after (blue line) the electrolysis.

Figure S5. SEM-EDX measurements of used glassy carbon electrodes after CPE.

Figure S6. Peak shift analysis in presence of AnsdH$^+$ under 1atm CO_2

Figure S7. CVs and FOWA plots for k_1 calculations.

Figure S8. Determination of rate dependance on [AnsdH$^+$] and [H_2O]

Figures S9. IR-SEC data to find the hydricity for (H-**1**)$^{3-}$

5. References

1. EXPERIMENTAL METHODS

Electrochemical Measurements. Cyclic voltammograms were recorded under a N_2 (99.998%, Praxair) atmosphere or CO_2 (from dry ice) in a standard 3-electrode configuration using a CH Instruments Electrochemical Analyzer model 620D or a model 1100B. A glassy carbon working electrode (CH Instruments, nominal surface area of 0.0707 cm^2) and a platinum wire electrode as counter electrode were used and the potential was applied versus a non-aqueous Ag/$AgNO_3$ (sat) reference electrode. Non-aqueous electrolyte solutions were stored over 3 Å molecular sieves which had been activated by heating under vacuum at 200°C for at least 72 hours. The average

ferrocenium/ferrocene half-wave potential measured in 0.1 M Bu_4NBF_4 CH_3CN solutions before and after each experiment was used to serve as an external standard (ferrocene) for the calibration of the non-aqueous reference electrode. The value for the ferrocenium/ferrocene half-wave potential was taken to be 0.4 V versus SCE.[1] Before each experiment, the glassy carbon working electrode was polished on a felt pad with alumina paste (0.05 micron, BASi), rinsed with water and acetone and dried with a Kimwipe unless otherwise stated. To obtain a reliable electron transfer rate constant, CVs were collected with 80% iR compensation.[2,3]

Controlled potential electrolysis (CPE) experiments were performed at a fixed potential in 25mL of solution containing catalyst (and organic acid or water if necessary) in a fritted H-cell using a glassy carbon plate with an area of 4 cm^2 (Tokai Carbon) as the working electrode, a coiled platinum wire (BASi) as the counter electrode, and the same reference electrode as that used in cyclic voltammetry experiments. All solutions were purged for about 20 min with 1 atm of N_2 or CO_2. For determination of the quantity of hydrogen produced, at the end of each experiment, a gaseous sample (0.1 mL) was drawn from the headspace via a Vici gastight syringe and injected into a gas chromatography−thermal conductivity detection system by Varian 3800 coupled with a Carboxen 1010 PLOT fused-silica column (30 m × 0.53 mm; Supelco) using N_2 (99.999%, Praxair) as the carrier gas. The H_2 concentration was determined using a working curve that was prepared from varied ratios of H_2 and N_2 gas. Quantification of formate was performed using ^{1}H NMR spectroscopy. An internal standard of a known amount of dimethylformamide (DMF) in C_6D_6, was prepared and sealed in a glass capillary tube. 500 μL of the CPE solution were injected into an NMR tube with the internal standard capillary. The integration of the ^{1}H resonance at 7.66 ppm for DMF, was used to quantify formate produced during CPE experiments (8.28 ppm). A plot of [HCOONa] vs the ratio of NMR integrals of DMF to formate was constructed from a series of NMR spectra collected of aqueous solutions of sodium formate and this plot was used to determine the concentration of formate produced in CPE experiments.

Infra-red spectra were recorded in a sealed liquid cell on a Bruker Alpha Infra-red spectrometer. IR-SEC (Infra-Red SpectroElectroChemistry) measurements were performed under 1 atm H_2 (g), using an optically transparent thin layer solution IR cell fabricated by Prof. Hartl at University of Reading at UK, as described previously.[4] In each experiment, electrochemical reduction of the species of interest was monitored by IR spectroscopy for a period of 0–20 min. Diffusion and mixing of the redox products, generated at the working and auxiliary electrodes in the IR cell was reasonably suppressed within the total experimental time.

Scanning Electron Microscopy and Energy Dispersion X-Ray Spectroscopic (SEM-EDX) Measurements. SEM-EDX data were acquired using S-4100T Hitachi HTA America coupled with Oxford INCA Energy. Accelerating voltage was set to 15 keV and quantization analysis were optimized before every analysis using pure copper metal. Characteristic X-ray was assigned to atoms using analytical grade standards: $CaCO_3$ for C; SiO_2 for O; Co metal for Co; and Cu metal for Cu.

Dynamic Light Scattering. Dynamic light scattering (DLS) was performed using the Zetasizer Nano ZS, Malvern instrument. The light source for the scattering experiments was a uniphase He-Ne laser emitting vertically polarized light at a wavelength of 633 nm. Data was collected at 25 °C with the scattering angle set to 90 degrees. The data was acquired and evaluated using the Zetasizer Nano software v3.30. All solvents used in the experiment were dried over 3 Å sieves and filtered through a 0.22-micron syringe filter before use. Samples for DLS were prepared in an air-tight 1 cm^2 quartz cuvette.

Preparation of Compounds. All manipulations were carried out using standard Schlenk or glovebox techniques under a dinitrogen atmosphere. Unless otherwise noted, solvents were deoxygenated and dried by thorough sparging with argon gas followed by passage through an activated alumina column. Anisidinium (*p*-OMePhNH$_2$), benzene sulfonamide (BSulf), 4-OMe benzene sulphonamide (^{OMe}BSulf) were purchased from Sigma Aldrich. Bu$_4$NBF$_4$,[5] and (*p*-OMePhNH$_3$)(BF$_4$),[6] were synthesized using previously reported procedures. The synthesis of (PhCH$_2$NMe$_3$)$_2$[Co$_{11}$C$_2$(CO)$_{23}$] (**1**$^{2-}$) was performed by following previously published method.[7]

2. CALCULATIONS

Calculation S1. Overpotential calculations for CO_2 reduction to formate by **1**$^{2-}$ in MeCN and in MeCN/H_2O solvent system.

Overpotential Definition

The overpotential (η) for a reaction is defined as the excess thermodynamic energy required to drive a reaction at a specific current density or activity.[3] The excess thermodynamic energy is the difference between applied potential (E_{app}) from the thermodynamic potential at standard state or equilibrium potential of the reaction under studied. For CO_2 reduction to formate (HCO_2^-) the overpotential (η) can be define as follows.

$$\eta = E^0(CO_2(g)/HCO_2^-)\ (s) - E_{applied} \qquad \text{(Equation S1)}$$

In heterogeneous catalysis $E_{applied}$ is anchored to another activity parameter such as fixed current density (10 mA cm^{-2}).

In homogenous catalysis,[8,9] $E_{applied}$ could be E_{onset} where rate of the reaction is very low or E_{cat} where reaction rate reaches maxima. Often, $E_{applied}$ is taken as $E_{cat/2}$ which signifies the overpotential required to reach half of maximum rate (see Illustration).

$$\eta = E^0(CO_2(g)/HCO_2^-)\ (s) - E_{cat/2} \qquad \text{(Equation S2)}$$

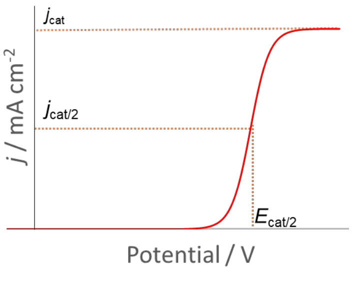

llustration: Definition of $E_{cat/2}$ based on CV traces collected under catalytic conditions. $E_{cat/2}$ is the point at which the catalytic wave reaches half of its maximum current ($j_{cat/2}$).

Another definition of overpotential in homogeneous electrocatalysis uses $E_{1/2}$ instead of $E_{cat/2}$ in equation S2. However, catalysis is not always observed near to the $E_{1/2}$ value of the molecular catalyst and so this definition does not always reflect the overpotential needed to drive the catalytic process.

In this work we used equation S2 with $E_{cat/2}$ to estimate the overpotential.

pK_a of proton sources

The pK_a of H_2CO_3 (17.01)[10,11,12] in CO_2 saturated MeCN/H_2O (95:5) mixture was used for overpotential calculation in this work.

The pK_a of anisidinium tetrafluoroborate (AnsdH$^+$) 11.86 in MeCN[13,14]

Calculation of Thermodynamic potential: $E^0(CO_2(g)/HCO_2^-)$ (s)

Thermodynamic potential for $H^+/2e^-$ reduction of CO_2 in MeCN solvent containing an organic acid was calculated using the equation published by Kubiak and coworkers:[15]

$$E^0(CO_2(g)/HCO_2^-)\ (s) = -0.77 - 0.030pK_a + 0.4 \text{ vs SCE} \qquad \text{(Equation S3)}$$

When AnsdH$^+$ (pK_a = 11.86 in MeCN) used as a proton source, the thermodynamic potential of CO_2 reduction to formate $E^0(CO_2(g)/HCO_2^-)$ (s) is -0.726 V vs SCE.

When H_2CO_3 (17.01) in CO_2 saturated MeCN/H_2O (95:5) mixture is used as a proton source, then Artero and coworkers have calculated that the thermodynamic potential of CO_2 reduction to formate $E^0(CO_2(g)/HCO_2^-)$ (s) is - 1.0 V vs SCE.[16]

Calculation of Overpotential

Using equation S2 to calculation overpotential.

In MeCN with AnsdH$^+$ $\quad \eta = E^0(CO_2(g)/HCO_2^-)\ (s) - E_{cat/2} = -0.726 - (-0.78) = 54$ mV

In CO_2 saturated MeCN/H_2O (95:5): $\eta = E^0(CO_2(g)/HCO_2^-)\ (s) - E_{cat/2} = -1.0 - (-1.01) = 10$ mV.

Calculation S2. Peak Shift Analysis for k_1 calculation.
To determine the PT rate constant, we employed observations specific to the EC-type mechanism.[17] The shift in peak position (E_p) upon variation of scan rate in the presence of an acid is given by equation S2

$$E_p = E^0 - \frac{0.78RT}{F} + \frac{RT}{2F}\ln\left(\frac{RTk_{PT1}}{Fv}\right) \qquad \text{(Equation S2)}$$

Equation S2 rearranged to equation S3

$$(E_p - E^0)\left(\frac{F}{RT}\right) = -0.78 + 0.5\ln\left(\frac{RT}{F}\right) + 0.5\ln\left(k_{PT1}\right) - 0.5\ln\left(\upsilon\right) \qquad \text{(Equation S3)}$$

For MeCN/H_2O (99.3:0.7) solvent system under 1 atm N_2. Plot of $(E_p - E^0)(\frac{F}{RT})$ vs ln(υ) results a straight line with -0.5 slope according to equation S3. From the intercept of best fit line of the plot, $k_{1,\,N2}$ was calculated. The calculations are shown here:

υ (V s^{-1})	ln(υ)	E_p (vs SCE)	$(Ep\text{-}E^0)(F/RT)$
0.2	-1.60944	-0.8488	4.09649
0.3	-1.20397	-0.85311	3.92873
0.4	-0.91629	-0.855	3.85506
0.5	-0.69315	-0.858	3.73824
0.6	-0.51083	-0.8615	3.60195
0.7	-0.35667	-0.86151	3.60156
0.8	-0.22314	-0.86469	3.47773
0.9	-0.10536	-0.86786	3.35429
1.2	0.18232	-0.86904	3.30834
1.4	0.33647	-0.87153	3.21119
2	0.69315	-0.87752	2.97829
3	1.09861	-0.88148	2.82393
4	1.38629	-0.88646	2.63001
Slope = -0.5 (R^2 = 0.988)<br>Intercept = -0.78 + 0.5 ln(RT/F) + 0.5ln(k_1) = 3.377<br>$k_1 = 1.5 \times 10^5$ s^{-1}<br>$k_1 = 3.9 \times 10^5$ $M^{-1}s^{-1}$; [H_2O] = 0.38 M			

For MeCN/H_2O (99.3:0.7) solvent system under 1 atm CO_2. Plot of $(E_p - E^0)(\frac{F}{RT})$ vs ln(υ) results a straight line, and the slope is fixed at -0.5 according to equation S3. From the intercept of best fit line of the plot, k_1 was calculated. The calculations are shown here:

υ (V s^{-1})	ln(υ)	E_p (vs SCE)	$(Ep\text{-}E^0)(F/RT)$
0.8	-0.22314	-0.81712	5.17435
1.2	0.18232	-0.82183	4.99094
1.6	0.47	-0.82493	4.87023
2	0.69315	-0.828	4.75068
3	1.09861	-0.83181	4.60218
4	1.38629	-0.83734	4.38698
5	1.60944	-0.84044	4.26627
6	1.79176	-0.84354	4.14555

Slope = -0.5 (R^2 = 0.998)
Intercept = -0.78 + 0.5 ln(RT/*F*) + 0.5ln(k_1) = 5.0917
$k_1 = 4.87 \times 10^6$ s^{-1}
$k_1 = 1.2 \times 10^7$ $M^{-1}s^{-1}$; [H_2O] = 0.38 M

For Anisidinium as acid source under 1 atm CO_2. Plot of $(E_p - E^0)(\frac{F}{RT})$ vs ln(ν) results a straight line, and the slope is fixed at -0.5 according to equation S3. From the intercept of best fit line of the plot, $k_{1,\,N2}$ was calculated. The calculations are shown here:

υ (V s^{-1})	ln(υ)	E_p (vs SCE)	(Ep-E^0)(F/RT)
0.2	-1.60944	-0.82591	4.83206
0.4	-0.91629	-0.84316	4.16035
0.6	-0.51083	-0.85177	3.82508
0.8	-0.22314	-0.8604	3.48902
1	0	-0.86714	3.22657
Slope = -0.8 (R^2 = 0.988)<br>Intercept = -0.78 + 0.5 ln(RT/*F*) + 0.5ln(k_1) = 3.577<br>$k_1 = 1.3 \times 10^5$ s^{-1}<br>$k_1 = 5 \times 10^8$ $M^{-1}s^{-1}$; [$AnsdH^+$] = 0.25 ×10^{-3} M			

FOW analysis for calculation of k_1

Foot-of-the-wave analysis (FOWA) was performed as described by Savéant and coworkers to calculate hydride formation rate (k_1).[18] The single sweep CV traces were used to parametrize the curve according to equation S4:

$$j = \frac{nF[\mathbf{1}^{4-}]k_1^{\frac{1}{2}}D_c^{\frac{1}{2}} \times 10^3}{1 + \exp\,[\frac{F}{RT}\left(E - E_{\frac{1}{2}}\right)]} \qquad \text{Equation S4}$$

Where, j is the current density (mA cm^{-2}) at the scanning potential E in V vs. SCE, $E_{1/2}$ = -0.96 V vs SCE, n = 2 for number of electrons, [$\mathbf{1}^{4-}$] is 1×10^{-8} mol cm^{-3}, and diffusion coefficient of $\mathbf{1}^{4-}$ (D)= 5.36×10^{-6} cm^2 s^{-1}, and $k_1 = k'[1^{4-}][H^+]$

From the linear portion of the plot between j vs $(1 + \exp\left[\frac{F}{RT}\left(E - E_{\frac{1}{2}}\right)\right])$, k_1 is calculated using proton concentration of 0.38 M.

Calculation S3. Calculation of K_1 from pK_a values.

Reaction of 1^{4-} With CO_2-saturated H_2O:

$\mathbf{1}^{4-} + H^+ \rightarrow (H\text{-}\mathbf{1})^{3-}$ $\quad 1/[pK_{a(H\text{-}\mathbf{1})}{}^{3-}] = -pK_{a(H\text{-}\mathbf{1})}{}^{3-} = 24.6$

$H_2CO_3 \rightarrow H^+ + HCO_3^-$ $\quad pK_{a\,(H2CO3)} = 23.4$

$\mathbf{1}^{4-} + H_2CO_3 \Leftrightarrow (H\text{-}\mathbf{1})^{3-} + HCO_3^-$

$\Delta G°_{PT} = 2.303RT(\Delta pK_a)$

$= 0.593 * 2.303[pK_{a(H2CO3)} - pK_{a(H\text{-}\mathbf{1})}{}^{3-}] = -1.63$ kcal/mol

With AnsdH⁺:

$\mathbf{1}^{4-} + H^+ \rightarrow (H\text{-}\mathbf{1})^{3-}$ $\quad 1/[pK_{a\,(H\text{-}\mathbf{1})}{}^{3-}] = -pK_{a\,(H\text{-}\mathbf{1})}{}^{3-} = 24.6$

$AnsdH^+ \rightarrow H^+ + Ansd$ $\quad pK_{a\,(AnsdH^+)} = 11.86$

$\mathbf{1}^{4-} + AnsdH^+ \Leftrightarrow (H\text{-}\mathbf{1})^{3-} + Ansd$

$\Delta G°_{PT} = 2.303RT(\Delta pK_a)$

$= 0.593 * 2.303[pK_{a(AnsdH^+)} - pK_{a(H\text{-}\mathbf{1})}{}^{3-}] = -17.3$ kcal/mol

$$K_1 = \frac{k_1}{k_{-1}} = e^{-\left(\frac{\Delta G^0_{PT}}{RT}\right)} \quad \text{(Equation S5)}$$

Calculation S4. Fast scan method for k_{obs} measurements

A fast scan method introduces by Azcarate, I. et al,[10] and Cometto, C. et al,[18] was used to calculate the observed rate constant (k_{obs}) for CO_2 reduction to formate by $\mathbf{1}^{3-}$. In fast scan method applying a very high scan rate results a scan rate independent region (pure kinetic regime) due to mutual compensation of catalyst diffusion and observed rate of catalysis.

The catalytic plateau current, j_c generated by a homogenous electrocatalyst at pure kinetic regime is defined as equation S6[19]:

$$j_c = nF[\text{cat}]k_{obs}^{\frac{1}{2}}D^{\frac{1}{2}} \times 10^3 \quad \text{(Equation S6)}$$

where, j_c is the background corrected plateau current density (mA cm^{-2}) at the scanning potential E, $n = 2$ for number of electrons for CO_2 reduction to formate, [Cat] is [$\mathbf{1}^{2-}$] in mol cm^{-3}, $D = 5.36 \times 10^{-6}$ cm^2 s^{-1}, k_{obs} is the observed rate constant (s^{-1}) and all other variables have been defined previously.

Parameters used to calculate the observed rate constant are:

In MeCN/H_2O (95:5): $j_c = 0.61$ mA cm^{-2}, [$\mathbf{1}^{2-}$] is 6×10^{-8} mol cm^{-3}, $k_{obs} = 525.9 \pm 20$ s^{-1}

In MeCN/AnsdH$^+$: $j_c = 1.67$ mA cm^{-2}, [$\mathbf{1}^{2-}$] is 2.2×10^{-7} mol cm^{-3}, $k_{obs} = 290 \pm 24$ s^{-1}

The k_{obs} values obtained were corrected for the measured FE which are 75% and 70% for formate under 1 atm CO_2, in MeCN/H_2O (95:5) and MeCN with added AnsdH$^+$, respectively.

Therefore, the values for k_{obs} 296 and 142 s^{-1}, respectively, in MeCN/H_2O (95:5) and MeCN with added $AnsdH^+$, under 1 atm CO_2.

Calculation S5. Experimental determination of pK_a and hydricity for (H-**1**)$^{3-}$.

Thermochemical insights such as hydricity (ΔG^0_{H-}) and pK_a for reactive intermediates (H-**1**)$^{3-}$ involved in catalytic cycle help with comparisons of performance to other catalysts. Hydricity is defined as the free energy for loss of hydride from a hydride donor. These thermochemical benchmarks also help to highlight the kinetic enhancements.

The hydricity for (H-**1**)$^{3-}$ was calculated using our previously published method.[21] Applying -1.2 V vs SCE in an infra-red spectroelectrochemical (IR-SEC) cell containing 0.2 mM **1**$^{3-}$ in 0.1 M Bu_4NBF_4 MeCN solution under 1 atm N_2 results a new species with ν_{CO} at 1941 (s), 1919 (b), 1893 (sh) and 1768 (w) cm^{-1} with concomitant disappearance of ν_{CO} at 1972 (s), 1890 (w), 1820 (sh) and 1800 (w) cm^{-1}. This change in the spectrum is associated with conversion of **1**$^{3-}$ into **1**$^{4-}$ and the isosbestic points at 1955, 1851 and 1787 cm^{-1} highlight that the conversion occurs without formation of side-products (Figure S9).

To determine the pK_a of (H-**1**)$^{3-}$, **1**$^{3-}$ was reduced to **1**$^{4-}$ in the IR-SEC cell in the presence of a 1 equivalent of benzene sulfonamide (BSulf; pK_a = 24.6 in MeCN) under 1 atm H_2 (Figure S9). The following thermochemical cycle describes the determination of the pK_a of (H-**1**)$^{3-}$ from the IR-SEC experiments (Scheme S1).

$$(\text{H-}\mathbf{1})^{3-} + \text{BSulf} \rightleftharpoons \mathbf{1}^{4-} + \text{H-BSulf}^{+} \qquad \text{p}K_{eq}$$

$$\text{H-BSulf}^{+} \rightleftharpoons \text{H}^{+} + \text{BSulf} \qquad \text{p}K_{acid}$$

$$(\text{H-}\mathbf{1})^{3-} + \text{BSulf} \rightleftharpoons \mathbf{1}^{4-} + \text{H}^{+}$$

$$\text{p}K_a \text{ of } (\text{H-}\mathbf{1})^{3-} = \text{p}K_{eq} + \text{p}K_{acid}$$

Scheme S1. Thermochemical cycle for calculation of pK_a of (H-1)$^{3-}$

The IR-SEC experiment in presence of BSulf indicates formation of new species with ν_{CO} at 1973, 1942 and 1892 cm^{-1}. The IR-SEC experiment was also performed in the presence of slightly stronger acid BuCOOH (pK_a = 22.7 in MeCN) and in that case H_2 gas formation was observed which suggests that the pK_a of (H-**1**)$^{3-}$ is above 22.7 in acetonitrile. When 5 equivalents of 4-methoxy benzene sulfonamide (^{OMe}BSulf; pK_a = 26.4 in MeCN) was used as acid no H_2 gas bubble formation was observed inside IR-SEC cell. The combination of these three experiments with the acids BuCOOH and ^{OMe}BSulf provides the lower and upper limit of pK_a of (H-**1**)$^{3-}$ and we estimate the pK_a value is 24.6 ± 1.

To calculate the ΔG_{H-} of (H-**1**)$^{3-}$ we can use the pK_a value along with some known thermochemical values,[15] as is illustrated in the thermochemical cycle in Scheme S2.

$$(\mathrm{H\text{-}}\mathbf{1})^{3-} + \mathrm{HA} \rightleftharpoons \mathbf{1}^{2-} + \mathrm{A}^- + \mathrm{H_2} \qquad 1.37{*}\mathrm{p}K_{eq}$$

$$\mathrm{H}^+ + \mathrm{A}^- \rightleftharpoons \mathrm{HA} \qquad -1.37{*}\mathrm{p}K_{acid}$$

$$\mathrm{H_2} \rightleftharpoons \mathrm{H}^+ + \mathrm{H}^- \qquad \Delta G_{H^-}(\mathrm{H_2})$$

$$(\mathrm{H\text{-}}\mathbf{1})^{3-} \rightleftharpoons \mathbf{1}^{2-} + \mathrm{H}^-$$

ΔG_{H^-} of $(\mathrm{H\text{-}}\mathbf{1})^{3-} = 1.37{*}\mathrm{p}K_{eq} - 1.37{*}\mathrm{p}K_{acid} + 76$ kcal/mol

Scheme S2. Thermochemical cycle for calculation of ΔG_{H^-} for (H-**1**)$^{3-}$

Hydricity for (H-**1**)$^{3-}$ is calculated using two limiting values of K_{eq} in scheme 2. In first case $K_{eq} > 10$ when H_2 formation is heavily favoured from the reaction between (H-**1**)$^{3-}$ and 1 eqv. added acid and in other case $K_{eq} < 0.1$ when H_2 formation is not observed. When 1 eqv. BSulf used as hydride acceptor, H_2 gas bubble was immediately observed inside IR-SEC cell. This suggests that in presence of BSulf $K_{eq} > 10$ and no H_2 gas observed when slightly less acidic ^{OMe}BSulf was used which implies that $K_{eq} < 0.1$. Using these two limiting values of $K_{eq,}$ we calculated that the hydricity value for (H-**1**)$^{3-}$ lies between 38.5 - 43.7; or 41.1 ± 2.6 kcal/mol.

Calculation S6. Theoretical hydricity value estimation for (H-**1**)$^{3-}$.

Theoretical hydricity value was calculated using corelation between experimental hydricity of metal hydride complexes ($\Delta G^0{}_{H-}$) and the first reduction potential of metal complexes ($E_{\frac{1}{2}}(M^{n+/(n-1)+})$) as described in the following equation S7 published by Kubiak and co-workers.[15]

$$\Delta G^0{}_{H^-} = BDFE + nFE_{\frac{1}{2}}(M^{n+/(n-1)+}) + \Delta G^0_{H\cdot/H^-} \qquad \text{Equation S7}$$

BDFE is homolytic bond dissociation free energy of metal hydride bond and $\Delta G^0_{H\cdot/H^-}$ (26.0 kcal/mol in acetonitrile) is free energy for reduction of a hydrogen atom to a hydride anion. Plot of $\Delta G^0{}_{H^-}$ vs $E_{\frac{1}{2}}(M^{n+/(n-1)+})$ of various transition metal complexes yield a linear correlation with slope equals to 23.06 kcal mol^{-1}V^{-1} (= nF) and intercept sum of BDEF and $\Delta G^0_{H\cdot/H^-}$

$$\Delta G^0{}_{H^-} = 23.06 * E_{\frac{1}{2}}(M^{n+/(n-1)+}) + 80.93\,; R^2 = 0.9105 \qquad \text{Equation S8}$$

It is worth to mentioned that the linear correlation between hydricity and $E_{\frac{1}{2}}(M^{n+/(n-1)+})$ implies that the homolytic bond dissociation energies of metal hydrides (BDFE) typically do not vary by much (less than 10 kcal/mol) across a series of complexes. Therefore, we assume a 10 kcal/mol error for estimated $\Delta G^0{}_{H^-}$.

Using $E_{\frac{1}{2}}(1^{4-/3-}) = -1.35\ V\ Fc/Fc^+$ in equation S8, the theoretical value for hydricity of H-**1**$^{3-}$ is $\Delta G^0{}_{H^-} = 49.8 \pm 10\ kcal/mol.$

3. TABLES

Table S1. Results from CPE experiments at -0.9 V (for CPE with $AnsdH^+$) -1.13 V (for CPE with 5% H_2O) with 0.1mM $\mathbf{1}^{2-}$ in 0.1 M Bu_4NBF_4 MeCN solution under 1 atm CO_2. [$AnsdH^+$] = 2.5 mM, and [H_2O] is 5% in MeCN.

Exp.	Acid Source	Time (min)	Charge passed (C)	FE (% H_2)	FE (% HCO_2^-)
$\mathbf{1}^{2-}$	$AnsdH^+$	10	1.34	25(3)	70(8)
R	$AnsdH^+$	10	0.67	45(4)	nd
none	$AnsdH^+$	10	0.53	45(3)	nd
$\mathbf{1}^{2-}$	H_2O	40	5.66	15(2)	75(5)
R	H_2O	40	4.1	68(4)	nd
none	H_2O	40	3.1	30(7)	nd

R represents trials when the electrode has been gently rinsed with MeCN and used in a subsequent CPE experiment in order to check for deposited, catalytically active material. Nd = not detected, detection limit is 0.0001 mM.

Table S2: Details of parameters used to construct the catalytic Tafel plots and LFER plots (Figures 5, 6) for selected molecular electrocatalysts.

Overpotential used in the Tafel plots are defined as $E_{cat/2}$ - $E_{CO2/HCOOH}$. All parameters in this table are determined from reported CV experiments. Note that some published Tafel-style plots in the literature include data that is a mix of CPE and CV data from various sources.

	$E_{cat/2}$ /V	$E_{CO2/HCOO^-}$ /V	$[CO_2]$ /M	k_{obs} /s^{-1}	FE(%)	Ref
$[Fe_4N(CO)_{12}]^-$: 95:5 MeCN:H_2O	-1.18	-1.0	0.24	0.6	97	20
$[Fe_4N(CO)_{11}(PPh_3)]^-$: 95:5 MeCN:H_2O	-1.45	-1.0	0.24	2.6	61	21
$[FeN_5Cl_2]^+$:DMF/H_2O	-1.25	-0.94	0.2	0.3[b]	80	22
$[FeP_4N_2]^{2+}$: MeCN/H_2O	-1.4	-1.0	0.24	2.6[b]	97.5	23
$CpCoP^{Cy}{}_2N^{Bn}{}_2I_2$: DMF, 1.1 M H_2O	-1.68	-1.05	0.2	400	90	16
$[Co(imino\text{-}bpy)]^{2+}$: MeCN/$H_2O$	-1.29	-1.0	0.24	11[b]	80	24
$Ir(POCOP^+)$: 0.1 M $NaHCO_3$ buffer, 1% v/v MeCN	-1.47	0.73	0.033	7.3	80	25
Ir(POCOP): 95:5 MeCN:H_2O	-1.55	-1.0	0.24	20	85	26
$[(bipy)Co(PyS)_2]^+$: MeCN/TFE	-1.34	-1.23	0.28	27.5	64	27
$[Pt(depe)]PF_6$: MeCN, $(CH_2)(TBD)_2$•H^+	-1.24	-1.23 @pKa 29.0	0.28	0.5	90	28
$[Co_{11}C_2(CO)_{23}]^{2-}$:MeCN/AnsdH^+	-0.76	-0.77	0.28	142	70	here
$[Co_{11}C_2(CO)_{23}]^{2-}$:95:5 MeCN:H_2O	-0.96	-1.0	0.24	296	75	here
$[Ni(qpdt)_2]^{1-}$:MeCN/TFE	-1.845	-1.4	0.28	89[b]	60	29
$[Mn(bpy)(CO)_3]^{1-}$/Fe-S	-1.45	-1.23	0.28	20[b]	88	30

All potential reported in this table vs SCE. [b] Rate were calculated from CPE data.

4. FIGURES

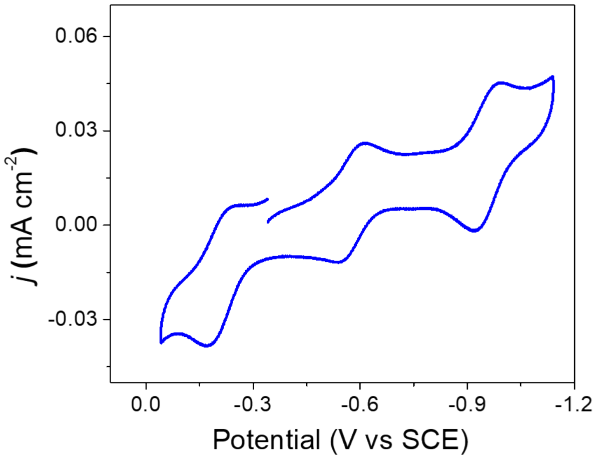

Figure S1. CV of 0.3 mM **1**$^{2-}$ recorded in 0.1 M Bu_4NBF_4 MeCN solution. Scan rate 50 mVs^{-1}. GC working electrode.

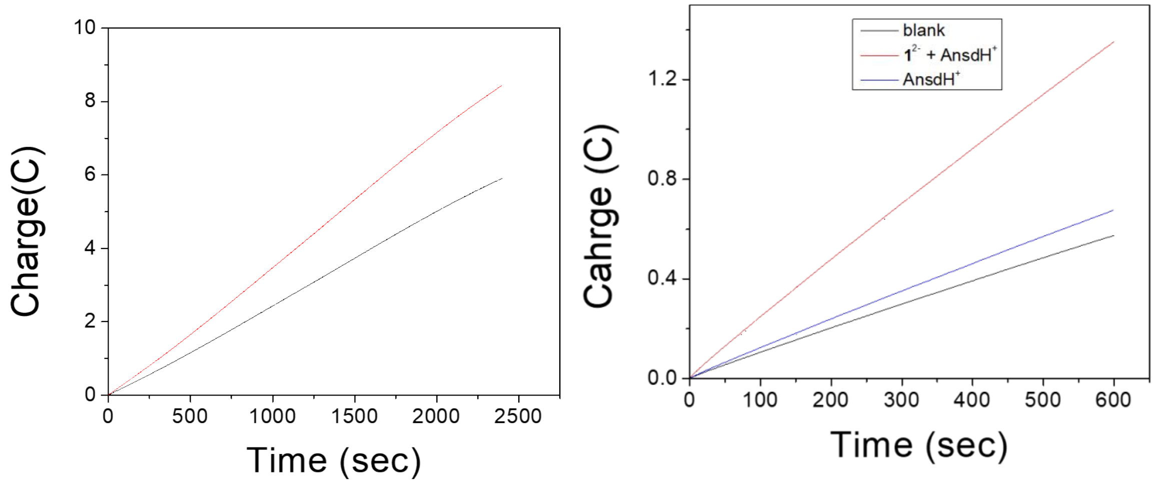

Figure S2A. (red) Charge vs. time plots from CPE performed at -1.13 V vs SCE over 40 min. Solution contains 0.1 M Bu_4NBF_4, 2.7 M H_2O (5%) with added 0.1 mM **1**$^{2-}$ under 1 atm CO_2. (black) Control experiment of CPE conducted with a used, rinsed electrode in a fresh electrolyte solution with no added catalyst under 1 atm CO_2. (right) Charge vs. time plots from CPE performed at -0.9 V vs SCE over 10 min. Solution contains 0.1 M Bu_4NBF_4, 25 mM $AnsdH^+$ with added 0.1 mM **1**$^{2-}$ under 1 atm CO_2. (Blue) Control experiment of CPE conducted with a used, rinsed electrode in a fresh electrolyte solution with no added catalyst under 1 atm CO_2.

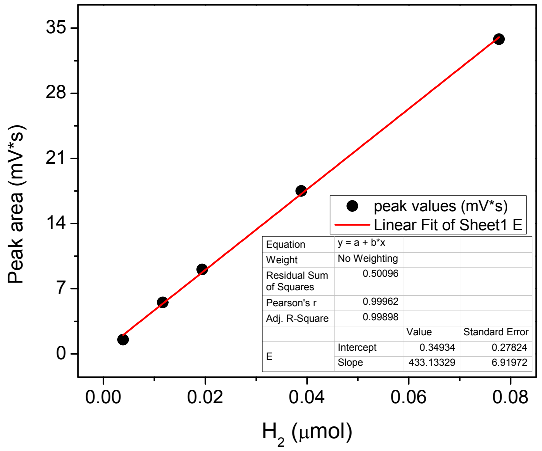

Figure S3A. Calibration curve used to quantify H_2 based on the GC-TCD data, following CPE experiments.

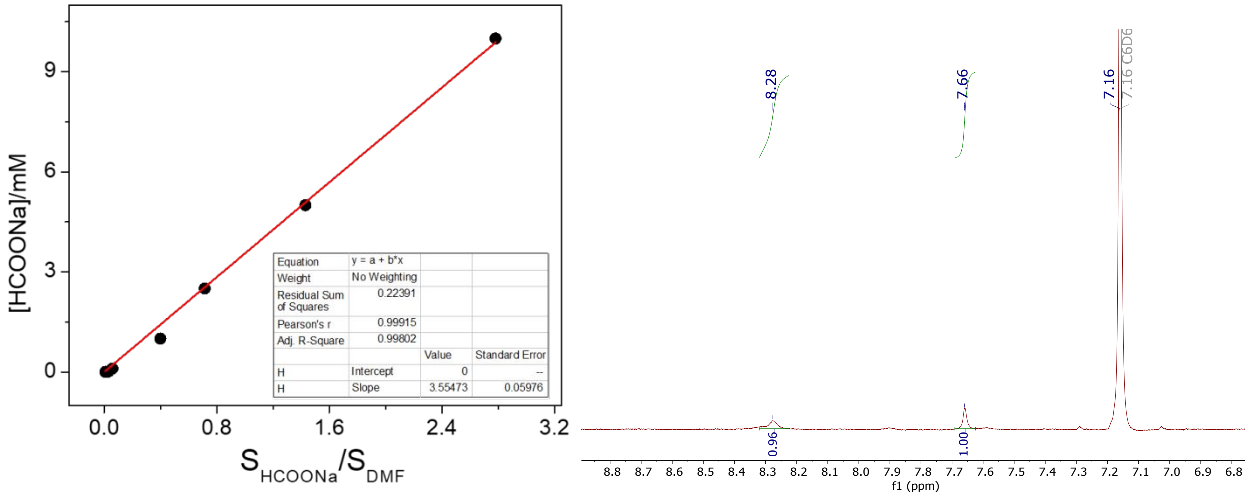

Figure S3B. (Left) ^{1}H NMR of electrolyte solution after CPE. Formate is observed at 8.28 ppm, a DMF internal standard is observed at 7.66 ppm. (right) Calibration curve used to quantify formate.

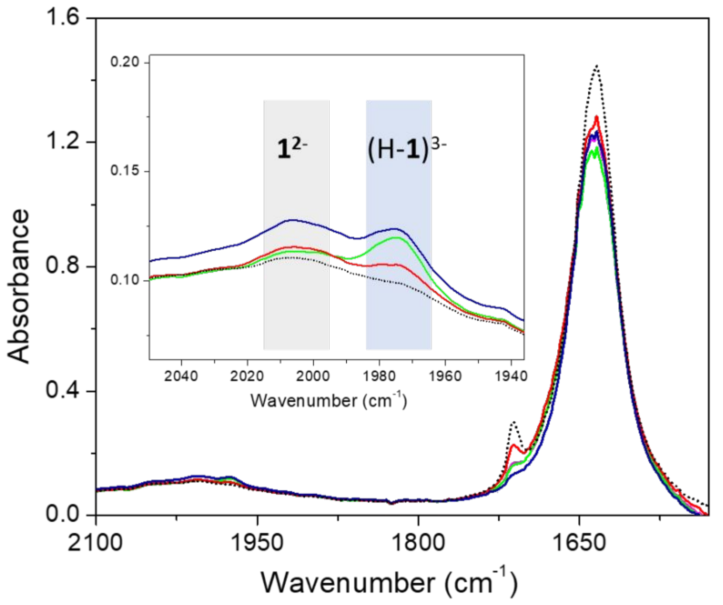

Figure S3C. Observation of formate (ν_{CO} = 1712 cm^{-1}) formation using IR spectroscopy during spectro-electrochemistry experiment performed in CO_2-saturated MeCN/H_2O (95:5) solution at -1.1 V vs. SCE. IR stretching frequency of formate in 0.1 M Bu_4NBF_4 MeCN under CO_2 is reported at 1608 and 1700 cm^{-1}.[30] Dotted line stands for 5 mM added formate in the same IR-SEC cell. Inset: zoomed view of IR spectra (2040-1940 cm^{-1}) collected at various time (blue: t = 1 min; green: t = 3 min; red: t = 7 min) which shows interconversion of (H-**1**)$^{3-}$ to **1**$^{2-}$ during experiment.

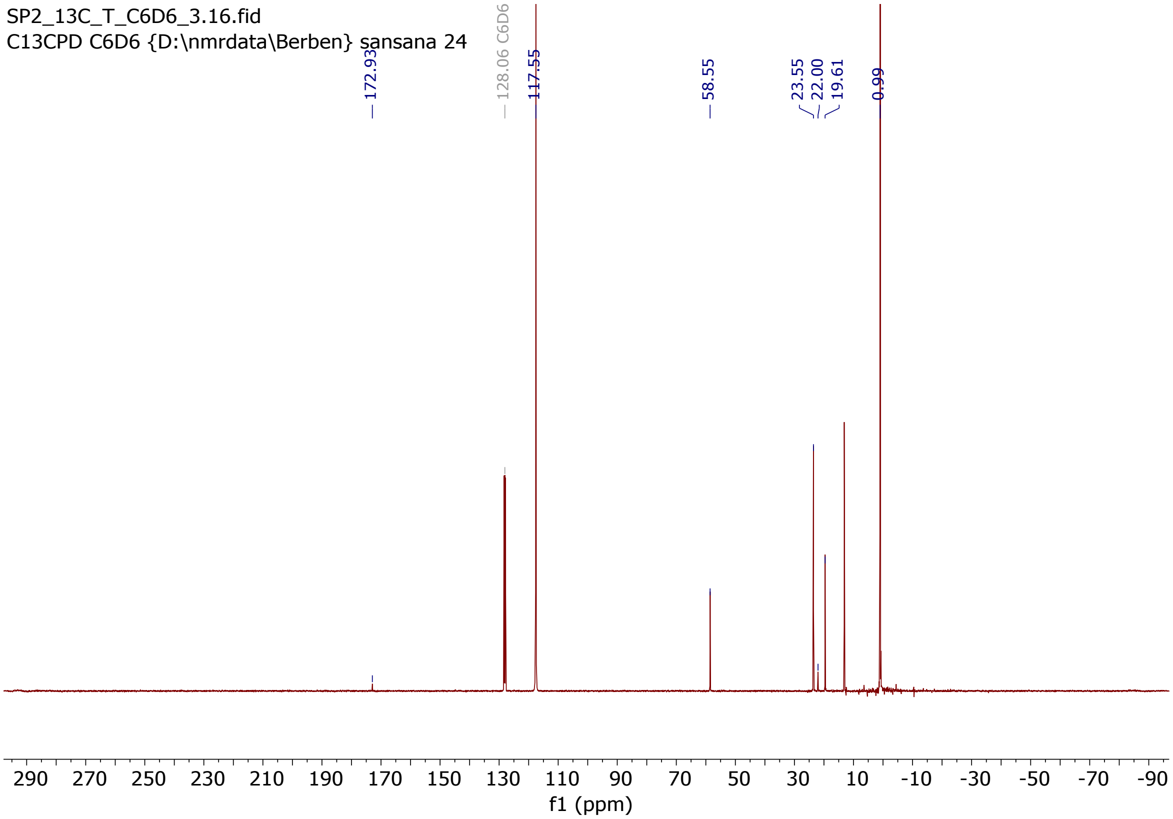

Figure S3D. $^{13}C\{^1H\}$ NMR of electrolyte solution after CPE with 0.1 mM $\mathbf{1}^{2-}$ and 2.8 M H_2O under $^{13}CO_2$ atmosphere. Formate is observed at 172.9 ppm, C_6D_6 is observed at 128.06 ppm. Peak at 117.55, 58.55, 23.55, 22.0, 19.6 and 0.99 ppm correspond to acetonitrile and tertabutyl ammonium cation from electrolyte solution respectively.

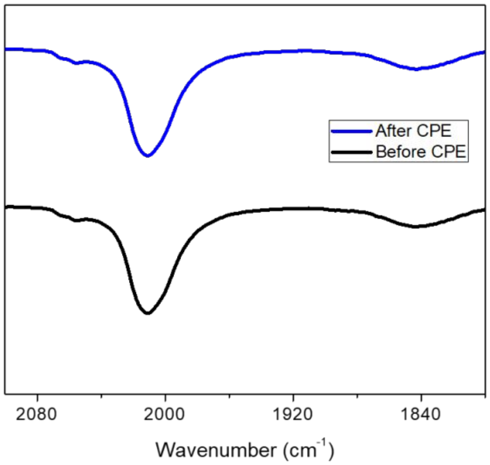

Figure S4. (left) IR spectra of 0.1 mM $\mathbf{1}^{2-}$ in 0.1 M Bu_4NBF_4 MeCN of a CPE solution before (black line) and after (blue line) the electrolysis. This shows that $\mathbf{1}^{2-}$ is stable during CPE.

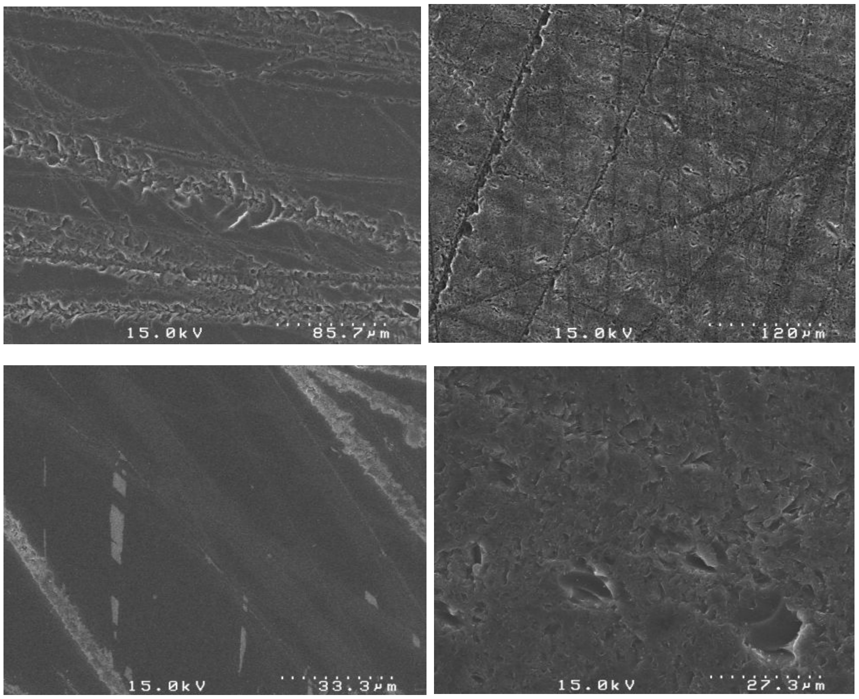

Figure S5A. SEM-EDX data was collected from the GC electrode before (top) and after (bottom) a CPE experiment under 1atm CO_2. (Left bottom) SEM image of GC electrode is collected from CPE solutions containing 0.1 mM **1**$^{2-}$ in 0.1 M Bu_4NBF_4 MeCN/H_2O (95:5) under 1 atm CO_2 over 40 min at -1.13 V. (right bottom) SEM image of GC electrode is collected from CPE solutions containing 0.1 mM **1**$^{2-}$ in 0.1 M Bu_4NBF_4 MeCN with added 25mM AnsdH$^+$ under 1 atm CO_2 over 10 min at -0.9 V.

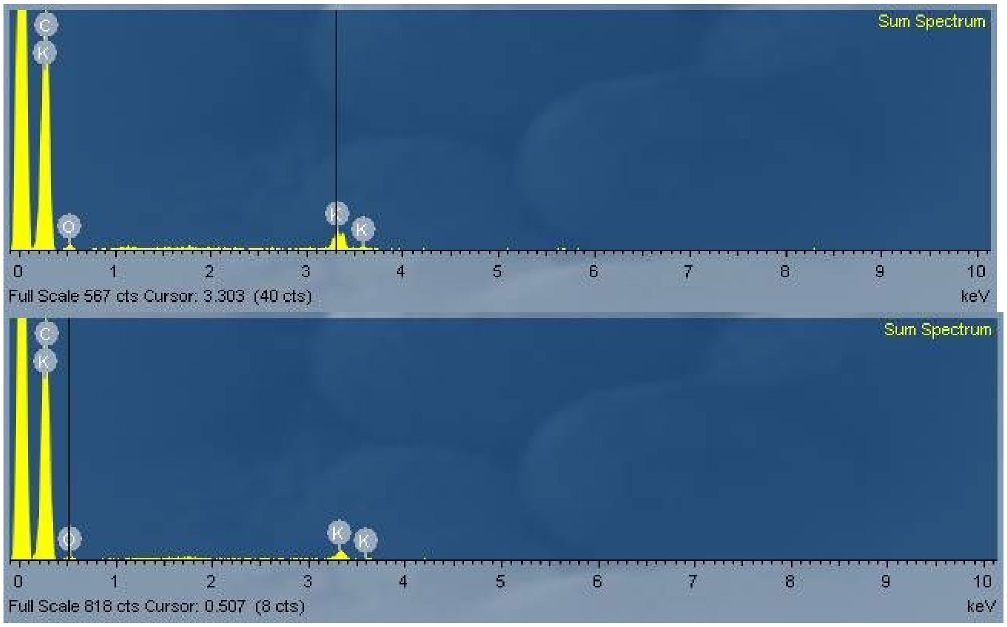

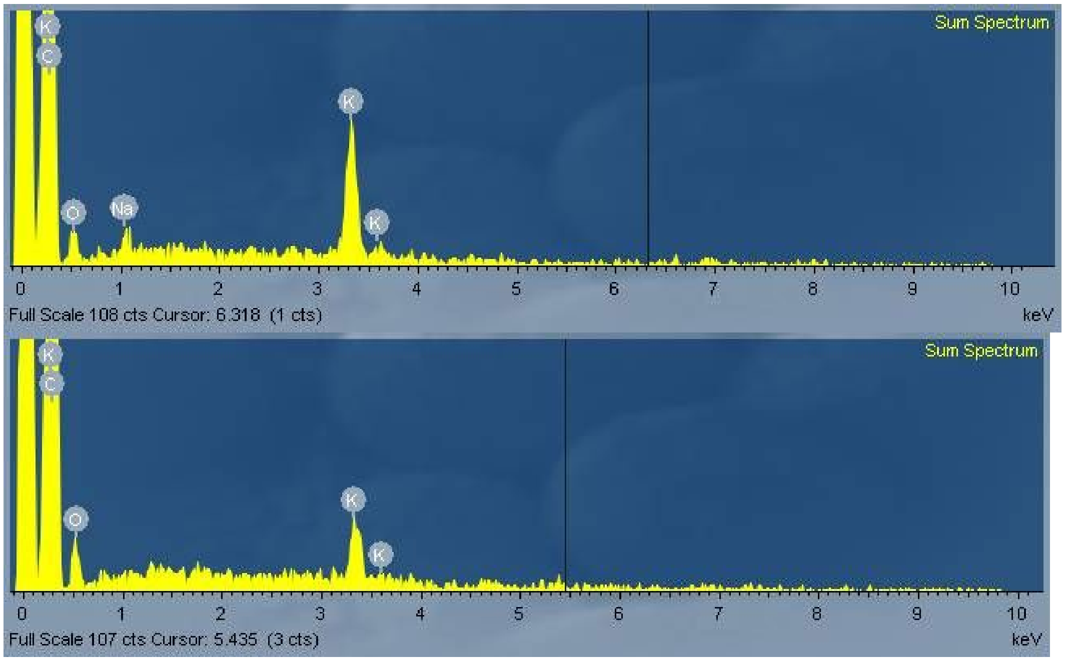

Figure S5B. (top two) EDX spectrum of polished glassy carbon plate. (bottom two) EDX spectrum of glassy carbon plate used for 40 min of CPE at -1.13V vs SCE in 0.1 mM **1**$^{2-}$, 2.7 M H_2O, 0.1 M Bu_4NBF_4 MeCN solution and EDX spectrum of glassy carbon plate used 10 min of CPE at -0.9 V vs. SCE in 0.1 mM **1**$^{2-}$, 25 mM AnsdH$^+$, 0.1 M Bu_4NBF_4 MeCN solution.

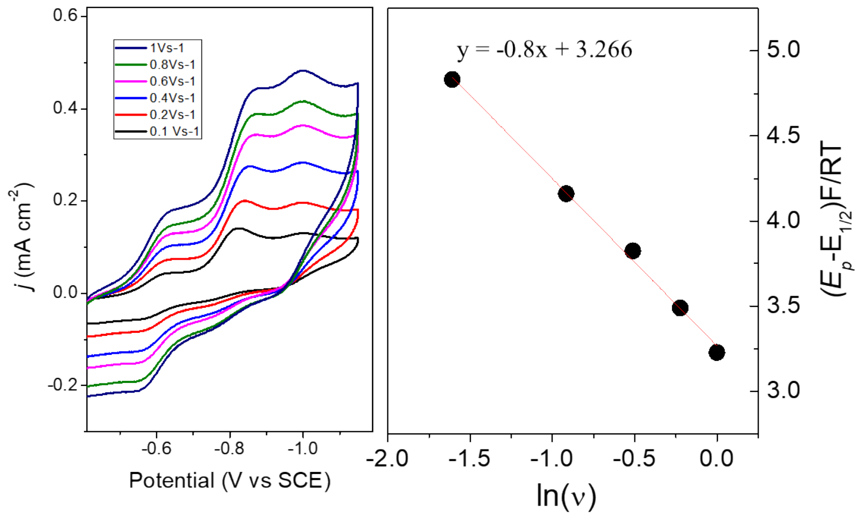

Figure S6. (left) CVs in 0.1 M Bu_4NBF_4 MeCN under CO_2 of 0.12 mM **1**$^{2-}$ at variable scan rates with 0.25 mM AnsdH$^+$. (right) Plot of $(E_p - E_{1/2})$(F/RT) vs ln(υ). The red line is a linear fit with negative slope of -0.8.

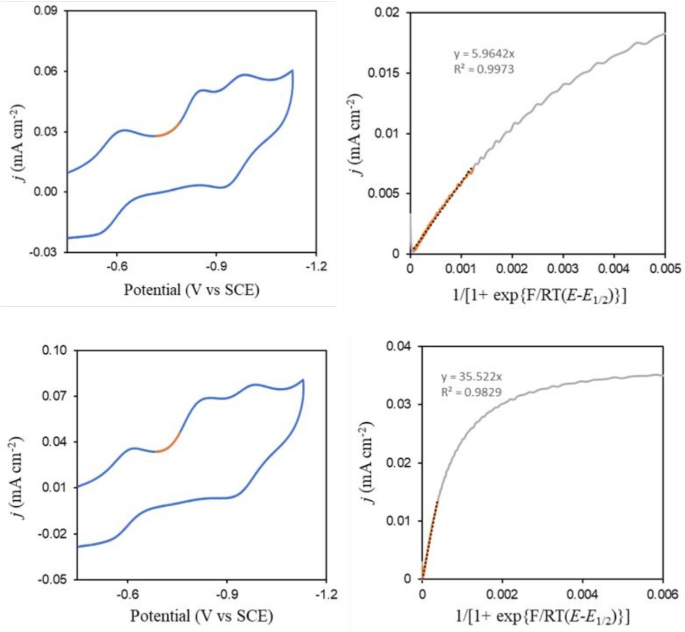

Figure S7. (Top left) CV of 0.1 mM **1**$^{2-}$ in 0.1 M Bu_4NBF_4 MeCN/H_2O (99.3:0.7) solution under 1 atm N_2, (bottom left) MeCN/H_2O (99.3:0.7) solution under 1 atm CO_2. Scan rate 0.1 V s^{-1}, GC working electrode. Orange highlight in CVs is FOW region used to calculate k_1. (Top and bottom right) Foot-of-the-wave analyses of the voltammogram in top left and bottom.

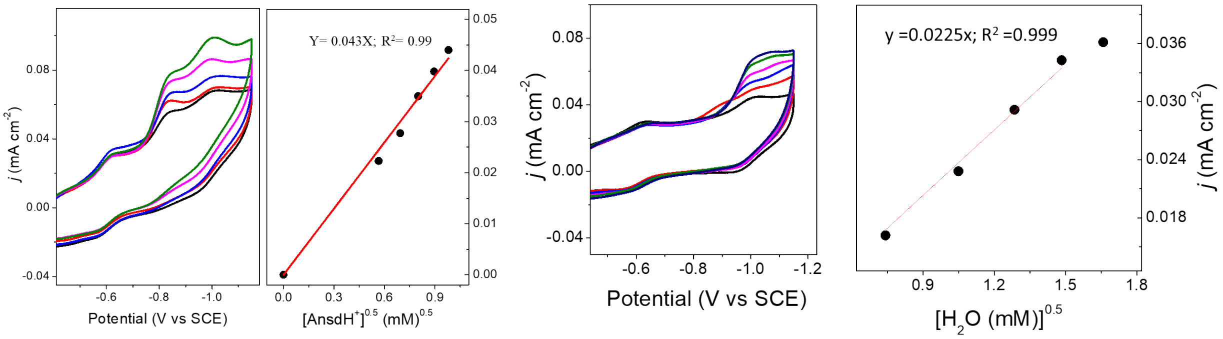

Figure S8. (left) CVs of 0.06 mM **1**$^{2-}$ in 0.1 M Bu_4NBF_4 MeCN under CO_2 with titration 0.32 (black), 0.48 (red), 0.64 (blue), 0.8 (pink), 0.96 mM (green) $AnsdH^+$. Plot of *j* vs. $[AnsdH^+]^{0.5}$ showing 1st order dependence of formate production on [$AnsdH^+$]. (right) CVs of 0.06 mM **1**$^{2-}$ in 0.1 M Bu_4NBF_4 MeCN under CO_2 with titration 0 (black), 0.55 (red), 1.1 (blue), 1.65 (pink), 2.2 mM (green) and 2.75 (violet) H_2O. Plot of *j* vs. $[H_2O]^{0.5}$ showing 1st order dependence of formate production on [H_2O].

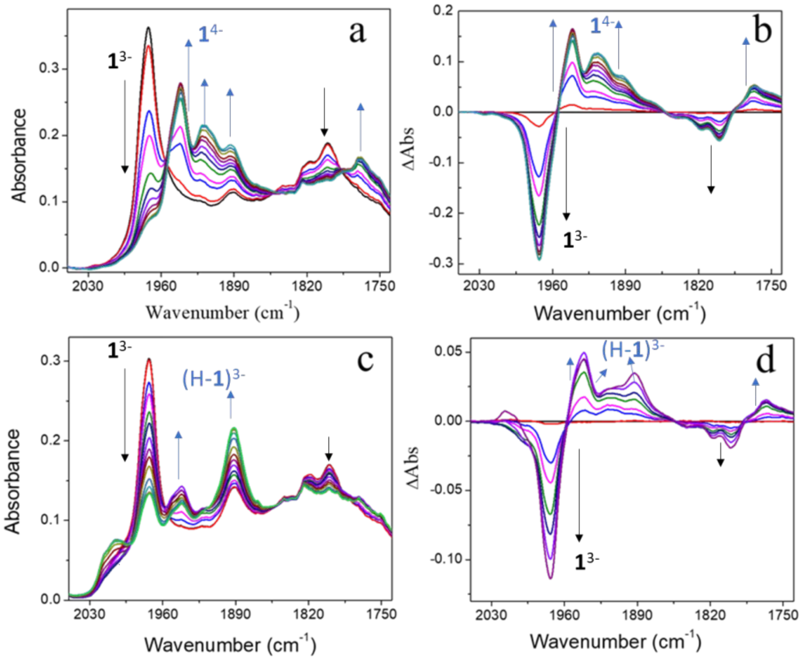

Figure S9. IR-SEC data collected to determine the hydricity for (H-**1**)$^{3-}$. The different colors represent successive IR spectra recorded at 2 min interval. (a) IR-SEC experiment showing conversion of 0.2 mM **1**$^{3-}$ to **1**$^{4-}$ in 0.1 MBu$_4$NBF$_4$ MeCN electrolyzed at -1.25 V vs. SCE (b) Difference absorbance spectra of plot for conversion of 0.2 mM **1**$^{3-}$ to **1**$^{4-}$ (c) IR-SEC experiment under 1 atm H$_2$ showing conversion of 0.2 mM **1**$^{3-}$ to **1**$^{4-}$ in 0.1 M Bu$_4$NBF$_4$ MeCN electrolyzed at -1.25 V vs. SCE with 1 equivalent (0.2 mM) of ^{OMe}BSulf and (d) Difference absorbance spectra showing conversion of 0.2 mM **1**$^{3-}$ to **1**$^{4-}$ in presence of 1eqv ^{OMe}BSulf under 1atm H$_2$. IR peaks associated with formation of (H-**1**)$^{3-}$ are highlighted by arrows in figure S9d.

5. REFERENCES

[1]Noviandri, I.; Brown, K. N.; Fleming, D. S.; Gulyas, P. T.; Lay, P. A.; Masters, A. F.; Phillips, L. The Decamethylferrocenium/Decamethylferrocene Redox Couple: A Superior Redox Standard to the Ferrocenium/Ferrocene Redox Couple for Studying Solvent Effects on the Thermodynamics of Electron Transfer. *J. Phys. Chem. B* **1999**, *103*, 6713–6722.

[2] Elgrishi, N.; Rountree, K. J.; McCarthy, B. D.; Rountree, E. S.; Eisenhart, T. T.; Dempsey, J. L. A Practical Beginner's Guide to Cyclic Voltammetry. *J. Chem. Educ.* **2018**, *95*, 197–206.

[3] A. J. Bard and L. R. Faulkner, *Electrochemical Methods: Fundamentals and Applications*, Johns Wiley and Sons, New York, 2nd edn, **2001**. pp22.

[4] Krejčik, M.; Daněk, M.; Hartl, F. Simple Construction of an Infrared Optically Transparent Thin-Layer Electrochemical Cell. Applications to the Redox Reactions of Ferrocene, $Mn_2(CO)_{10}$ and $Mn(CO)_3$(3,5-Di-t-Butyl-Catecholate)-. *J. Electroanal. Chem.* **1991**, *317*, 179–187.

[5] House, H. O.; Feng, E.; Peet, N. P. A Comparison of Various Tetraalkylammonium Salts as Supporting Electrolytes in Organic Electrochemical Reactions. *J. Org. Chem.* **1971**, *36*, 2371–2375.

[6] McCarthy, B. D.; Martin, D. J.; Rountree, E. S.; Ullman, A. C.; Dempsey, J. L. Electrochemical Reduction of Brønsted Acids by Glassy Carbon in Acetonitrile-Implications for Electrocatalytic Hydrogen Evolution. *Inorg. Chem.* **2014**, *53*, 8350–8361.

[7] (a) Pattanayak, S.; Berben, L. A. Cobalt Carbonyl Clusters Enable Independent Control of Two Proton Transfer Rates in the Mechanism for Hydrogen Evolution. *ChemElectroChem* **2021**, *8*, 2488–2494; (b) Ciabatti, I.; Femoni, C.; Hayatifar, M.; Iapalucci, M. C.; Longoni, G.; Pinzino, C.; Solmi, M. V.; Zacchini, S. The Redox Chemistry of $[Co_6C(CO)_{15}]^{2-}$: A Synthetic Route to New Co-Carbide Carbonyl Clusters. *Inorg. Chem.* **2014**, *53*, 3818–3831.

[8] Stratakes, B. M.; Dempsey, J. L.; Miller, A. J. M. Determining the Overpotential of Electrochemical Fuel Synthesis Mediated by Molecular Catalysts: Recommended Practices, Standard Reduction Potentials, and Challenges. *ChemElectroChem* **2021**, 1–21.

[9] Appel, A. M.; Helm, M. L. Determining the Overpotential for a Molecular Electrocatalyst. *ACS Catal.* **2014**, *4*, 630–633.

[10] Azcarate, I.; Costentin, C.; Robert, M.; Savéant, J. M. Through-Space Charge Interaction Substituent Effects in Molecular Catalysis Leading to the Design of the Most Efficient Catalyst of CO_2-to-CO Electrochemical Conversion. *J. Am. Chem. Soc.* **2016**, *138*, 16639–16644.

[11] Costentin, C.; Drouet, S.; Robert, M.; Savéant, J.-M. A Local Proton Source Enhances CO_2 Electroreduction to CO by a Molecular Fe Catalyst. *Science* **2012**, *338*, 90−94.

[12] Azcarate, I.; Costentin, C.; Robert, M.; Savéant, J. M. Dissection of Electronic Substituent Effects in Multielectron-Multistep Molecular Catalysis. Electrochemical CO_2-to-CO Conversion Catalyzed by Iron Porphyrins. *J. Phys. Chem. C* 2016, *120*, 28951–28960.

[13] Kaljurand, I.; Kutt, A.; Soovali, L.; Rodima, T.; Maemets, V.; Leito, I.; Koppel, I. A. Extension of the Self-Consistent Spectrophotometric Basicity Scale in Acetonitrile to a Full Span of 28 pKa Units: Unification of Different Basicity Scales. *J. Org. Chem.* **2005**, *70*, 1019– 1028

[14] Roberts, J. A. S.; Bullock, R. M. Direct Determination of Equilibrium Potentials for Hydrogen Oxidation/Production by Open Circuit Potential Measurements in Acetonitrile. *Inorg. Chem.* 2013, *52*, 3823–3835.

[15] Waldie, K. M.; Ostericher, A. L.; Reineke, M. H.; Sasayama, A. F.; Kubiak, C. P. Hydricity of Transition-Metal Hydrides: Thermodynamic Considerations for CO2 Reduction. *ACS Catal.* **2018**, *8*, 1313–1324.

[16] Roy, S.; Sharma, B.; Pécaut, J.; Simon, P.; Fontecave, M.; Tran, P. D.; Derat, E.; Artero, V. Molecular Cobalt Complexes with Pendant Amines for Selective Electrocatalytic Reduction of Carbon Dioxide to Formic Acid. *J. Am. Chem. Soc.* **2017**, *139*, 3685–3696.

[17] Roubelakis, M. M.; Bediako, D. K.; Dogutan, D. K.; Nocera, D. G. Proton-Coupled Electron Transfer Kinetics for the Hydrogen Evolution Reaction of Hangman Porphyrins. *Energy Environ. Sci.* **2012**, *5*, 7737–7740.

[18] Cometto, C.; Chen, L.; Anxolabéhère-Mallart, E.; Fave, C.; Lau, T.-C.; Robert, M. Molecular Electrochemical Catalysis of the CO_2-to-CO Conversion with a Co Complex: A Cyclic Voltammetry Mechanistic Investigation. *Organometallics* **2019**, *38*, 1280– 1285.

[19] Savéant J. -M., *Elements of Molecular and Biomolecular Electrochemistry*, John Wiley & Sons, Hoboken, **2006**. pp108-114.

[20] Taheri, A.; Carr, C. R.; Berben, L. A. Electrochemical Methods for Assessing Kinetic Factors in the Reduction of CO_2 to Formate: Implications for Improving Electrocatalyst Design. *ACS Catal.* **2018**, *8*, 5787–5793.

[21] Loewen, N. D.; Thompson, E. J.; Kagan, M.; Banales, C. L.; Myers, T. W.; Fettinger, J. C.; Berben, L. A. A Pendant Proton Shuttle on [Fe4N(CO)12]- Alters Product Selectivity in Formate vs. H_2 Production via the Hydride $[H\text{-}Fe_4N(CO)_{12}]^-$. *Chem. Sci.* **2016**, *7*, 2728–2735.

[22] Chen, L.; Guo, Z.; Wei, X.; Gallenkamp, C.; Bonin, J.; Lau, K.; Lau, T.; Robert, M. Molecular Catalysis of the Electrochemical and Photochemical Reduction of CO_2 with Earth-Abundant Metal Complexes. Selective Production of CO vs HCOOH by Switching of the Metal Center. *J. Am. Chem. Soc.* **2015**, *137*, 10918−10921.

[23] Bi, J.; Hou, P.; Liu, F.; Kang, P. Electrocatalytic Reduction of CO_2 to Methanol by Iron Tetradentate Phosphine Complex Through Amidation Strategy. *ChemSusChem* **2019**, *12*, 2195 –2201.

[24] Liu, F.; Bi, J.; Sun, Y.; Luo, S.; Kang, P. Cobalt Complex with Redox-Active Imino Bipyridyl Ligand for Electrocatalytic Reduction of Carbon Dioxide to Formate. *ChemSusChem* **2018**, *11*,1656 –1663.

[25] Kang, P.; Meyer, T. J.; Brookhart, M. Selective Electrocatalytic Reduction of Carbon Dioxide to Formate by a Water-Soluble Iridium Pincer Catalyst. *Chem. Sci.* **2013**, *4*, 3497–3502.

[26] Kang, P.; Cheng, C.; Chen, Z.; Schauer, C. K.; Meyer, T. J.; Brookhart, M. Selective Electrocatalytic Reduction of CO_2 to Formate by Water-Stable Iridium Dihydride Pincer Complexes. *J. Am. Chem. Soc.* **2012**, *134*, 5500–5503.

[27] Dey, S.; Todorova, T. K.; Fontecave, M.; Mougel, V. Electroreduction of CO_2 to Formate with Low Overpotential Using Cobalt Pyridine Thiolate Complexes. *Angew. Chemie* **2020**, *132*, 15856–15863.

[28] (a). Cunningham, D. W.; Yang, J. Y. Kinetic and Mechanistic Analysis of a Synthetic Reversible CO_2/HCO_2^-Electrocatalyst. *Chem. Commun.* **2020**, *56*, 12965–12968; (b). Cunningham, D. W.; Barlow, J. M.; Velazquez, R. S.; Yang, J. Y. Reversible and Selective CO_2 to HCO_2^- Electrocatalysis near the Thermodynamic Potential. *Angew. Chemie - Int. Ed.* **2020**, *59*, 4443–4447.

[29] Fogeron, T.; Todorova, T. K.; Porcher, J.; Gomez-mingot, M.; Chamoreau, L.; Mellot-draznieks, C.; Li, Y.; Fontecave, M. A Bioinspired Nickel(Bis-Dithiolene) Complex as a Homogeneous Catalyst for Carbon Dioxide Electroreduction. *ACS Catal*. **2018**, *8*, 2030−2038.

[30] Dey, S.; Masero, F.; Brack, E.; Fontecave, M.; Mougel, V. Electrocatalytic Metal Hydride Generation Using Concerted Proton Electron Transfer Mediators. *Nature* **2022**, *607*, 499-506.
